# Molecular characterization of SARS-CoV-2 Omicron clade and clinical presentation in children

**DOI:** 10.1038/s41598-024-55599-0

**Published:** 2024-03-04

**Authors:** Rossana Scutari, Valeria Fox, Vanessa Fini, Annarita Granaglia, Anna Chiara Vittucci, Andrea Smarrazzo, Laura Lancella, Francesca Calo’ Carducci, Lorenza Romani, Laura Cursi, Paola Bernaschi, Cristina Russo, Andrea Campana, Stefania Bernardi, Alberto Villani, Carlo Federico Perno, Claudia Alteri

**Affiliations:** 1 Multimodal Laboratory Research Unit, Bambino Gesù Children’s Hospital, IRCCS, Rome, Italy; 2 Department of Oncology and Hemato-Oncology, University of Milan, Milan, Italy; 3 Major School in Microbiology and Virology, Campus Bio-Medico University, Rome, Italy; 4 Academic Department of Pediatrics, Bambino Gesù Children’s Hospital, IRCCS, Rome, Italy; 5 Microbiology and Diagnostics in Immunology, Bambino Gesù Children’s Hospital, IRCCS, Rome, Italy

**Keywords:** SARS-CoV-2 genomic evolution, Omicron clade, Minority variants, COVID-19 in children, SARS-CoV-2, Viral evolution

## Abstract

Since its emergence, SARS-CoV-2 Omicron clade has shown a marked degree of variability and different clinical presentation compared with previous clades. Here we demonstrate that at least four Omicron lineages circulated in children since December 2021, and studied until November 2022: BA.1 (33.6%), BA.2 (40.6%), BA.5 (23.7%) and BQ.1 (2.1%). At least 70% of infections concerned children under 1 year, most of them being infected with BA.2 lineages (n = 201, 75.6%). Looking at SARS-CoV-2 genetic variability, 69 SNPs were found to be significantly associated in pairs, (phi <  − 0.3 or > 0.3 and p-value < 0.001). 16 SNPs were involved in 4 distinct clusters (bootstrap > 0.75). One of these clusters (A23040G, A27259C, T23617G, T23620G) was also positively associated with moderate/severe COVID-19 presentation (AOR [95% CI] 2.49 [1.26–4.89] p-value: 0.008) together with comorbidities (AOR [95% CI] 2.67 [1.36–5.24] p-value: 0.004). Overall, these results highlight the extensive SARS-CoV-2 Omicron circulation in children, mostly aged < 1 year, and provide insights on viral diversification even considering low-abundant SNPs, finally suggesting the potential contribution of viral diversification in affecting disease severity.

## Introduction

Along the pandemic course, Severe Acute Respiratory Syndrome COronaVirus 2 (SARS-CoV-2) has evolved rapidly, accumulating single nucleotide polymorphisms (SNPs) and generating new variants characterized by different transmissibility, virulence, and immune evasion^1–8^. Some of these have been declared by WHO to be of particular concern due to their high transmissibility and impact on the general population.

Starting from the end of 2021, the Omicron clade has out-competed previous variants, rapidly becoming the dominant one. Since its emergence until the present, Omicron has undergone substantial genetic evolution, as evidenced by the identification of different lineages (BA.1, BA.2, BA.3, BA.4, BA.5) and their descendant and recombinant lines (XD, XE, XF, BQ etc.)^9–12^. These lineages share the same ancestor with the first, but at the same time are characterized by unique mutational patterns acquired over evolution.

In this regard, several studies have shown that the new amino acidic mutations at the spike protein characterizing Omicron clade increased transmissibility and evasion to different neutralizing antibodies (NAbs)^13–16^. Nonetheless, these mutations have not worsened clinical presentation, being frequently associated with less severe manifestations in the adult population^17–19^. In children, the dynamics of SARS-CoV-2 evolution are poorly studied, as is the potential clinical impact of unique mutational profiles. In our previous work, we demonstrated a sizeable circulation of different SARS-CoV-2 lineages in SARS-CoV-2 positive patients aged ≤ 12 years over the first four pandemic waves (from pandemic start to delta clade), but no significant associations were found between lineages and COVID-19 presentation, even if a lower number of moderate/severe cases were found during alpha-clade epidemic^20^.

It is well known that, in the evolutionary pathway, new advantageous mutations are selected and can become dominant. In line with this, restricting the study of the SARS-CoV-2 genome only to the level of consensus sequences may limit the complete knowledge and understanding of the evolutionary pathways of the virus, due to the presence of non-constitutive and low abundant mutations that can expand significantly over time and might cause alteration in COVID-19 manifestation^21^. Consequently, the early detection of these low-abundant mutations could warn, as well as predict, their selection as constitutive and highly abundant mutations in upcoming variants^21^.

Considering these premises, in the present study, we integrated epidemiological, viral genetic, and clinical data to characterize SARS-CoV-2 Omicron infection in a cohort of paediatric patients, referred to Bambino Gesù Children Hospital in Rome.

## Results

### Patients’ characteristics

From late December 2021 to early November 2022, 2831 new SARS-CoV-2 diagnoses were performed in pediatric subjects (< 12-year-old) at the Bambino Gesù Children Hospital IRCCS in Rome. For 1182 diagnoses, nasopharyngeal swabs characterized by cycle threshold (Ct) < 29 or Antigenic Cut off index (COI) > 1000 and demographic and clinical information were available and retrieved. Whole SARS-CoV-2 genome was performed for 713 randomly selected samples and was successfully obtained for 657 final samples. Demographic and clinical characteristics are reported in Table 1.

**Table 1:** Demographic and clinical characteristics of the 657 SARS-CoV-2-infected patients against Omicron lineages.

	Overall	BA.1	BA.2	BA.5	BQ.1	p-value
	N = 657	N = 221	N = 266	N = 156	N = 14	
Demographic characteristics						
Age, years	0.56 (0.23–1.66)	0.67 (0.22–2.62)	0.50 (0.22–0.95)	0.63 (0.29–1.50)	0.25 (0.15–1.11)	0.093
< 1	449 (68.3)	135 (61.1)	201 (75.6)	104 (66.7)	9 (64.3)	0.007
1–5.5	148 (22.5)	58 (26.2)	42 (15.8)	43 (27.6)	5 (35.7)	0.006
> 5.5	60 (9.1)	28 (12.7)	23 (8.6)	9 (5.8)	0 (0.0)	0.074
Sex, male	347 (52.8)	124 (56.1)	137 (51.5)	79 (50.6)	7 (50.0)	0.686
Origin						
Caucasian	586 (89.2)	198 (89.6)	239 (89.8)	136 (87.2)	13 (92.9)	0.800
Asian	34 (5.2)	11 (5.0)	10 (3.8)	12 (7.7)	1 (7.1)	0.358
North American	12 (1.8)	4 (1.8)	6 (2.3)	2 (1.3)	0 (0.0)	0.851
Latin America	9 (1.4)	2 (0.9)	3 (1.1)	4 (2.6)	0 (0.0)	0.511
African	16 (2.4)	6 (2.7)	8 (3.0)	2 (1.3)	0 (0.0)	0.645
Residency						
Lazio	586 (89.2)	195 (88.2)	240 (90.2)	139 (89.1)	12 (85.7)	0.878
Others^a^	71 (10.8)	26 (11.8)	26 (9.8)	17 (10.9)	2 (14.3)	0.878
Comorbidity	123 (18.7)	50 (22.6)	43 (16.2)	27 (17.3)	3 (21.4)	0.305
First diagnosis	31 Mar 22 (09 Feb 22–03 Jun 22)	31 Jan 22 (21Jan 22–12 Feb 22)	06 Apr 22 (23 Mar 22–24 Apr 22)	28 Jul 22 (15 Jul 22–12 Aug 22)	23 Oct 22 (15 Oct 22–03 Nov 22)	< 0.001
Clinical characteristics						
Symptoms at admission						
Mild^b^	539 (82.0)	166 (75.1)	222 (83.5)	140 (89.7)	11 (78.6)	0.003
Moderate/severe^c^	54 (8.2)	33 (14.9)	17 (6.4)	3 (1.9)	1 (7.1)	< 0.001
Asymptomatic	64 (9.7)	22 (10.0)	27 (10.2)	13 (8.3)	2 (14.3)	0.863
Hospitalization	97 (14.8)	42 (19.0)	38 (14.3)	15 (9.6)	2 (14.3)	0.090
SARS-CoV-2 rtPCR^d^						
Mean cycle thresholds	18 (15–20)	18 (16–21)	17 (15–20)	17 (15–19)	–	0.013
E	17 (15–20)	18 (15–21)	17 (15–19)	16 (15–18)	–	< 0.001
RdRp	20 (17–25)	21 (17–26)	21 (17–24)	19 (16–22)	–	0.669
N/N2	18 (16–21)	19 (16–22)	18 (16–21)	18 (16–20)	–	0.091
S	18 (15–21)	17 (14–20)	18 (16–22)	19 (16–22)	–	0.521
Orf1ab	19 (16–23)	19 (15–21)	20 (17–24)	20 (16–25)	–	0.518
SARS-CoV-2 Ag^e^	5097 (2395–10,869)	–	–	5000 (2267–10,501)	5756 (4611–11,160)	0.373

Data are expressed as median (IQR), or N (%).

Two-sided p-values were calculated by Kruskal–Wallis test, or Chi-square test for trend, as appropriate.

^a^Other includes Abruzzo (n = 6), Albania (n = 1), Basilicata (n = 1), Calabria (n = 6), Campania (n = 11), Emilia-Romagna (n = 1), France (n = 1), Friuli-Venezia-Giulia (n = 1), Germany (n = 1), Liguria (n = 1), Lombardy (n = 3), Marche (n = 4), Molise (n = 2), Piemonte (n = 1), Puglia (n = 7), Romania (n = 4), Sardegna (n = 2), Sicilia (n = 3), Switzerland (n = 1), Toscana (n = 4), Ukraine (n = 7), Umbria (n = 3).

^b^Including: symptoms of upper respiratory airways (rinhitis, pharyngo-adenitis, laryngitis) and/or gastrointestinal symptoms.

^c^Including: symptoms of lower respiratory airways (pneumonia, bronchitis and bronchiolitis) with or without gastrointestinal symptoms.

^d^Real-time reverse transcription PCR Ct (cycle threshold) values were obtained by AllplexTM 2019-nCoV Assay Seegene (target E, RdRp, N), and Xpert Xpress SARS-CoV-2 Assay, Cepheid (Target E and N2).

^e^SARS-CoV-2 Ag COI (Cut Off Index) values were obtained by Roche SARS-CoV-2 Rapid Antigen Test and SD Biosensor COVID-19 Ag FIA.

Three hundred and forty-seven (52.8%) individuals were male. The median age was 0.56 (interquartile range [IQR] 0.23–1.66) years. Four hundred and forty-nine (68.3%) individuals were under 1 year of age. Most individuals lived in Lazio region (n = 586, 89.2%), and were Caucasian (n = 586, 89.2%). At the time of testing, mild infections were the most prevalent (539 cases, 82.0%), followed by asymptomatic infections (64, 9.8%). Only 8.2% of patients (n = 54) had a moderate/severe infection. Ninety-seven patients required hospitalization (97, 14.8%).

### Distribution of SARS-CoV-2 lineages affecting paediatric population

**Figure 1:**
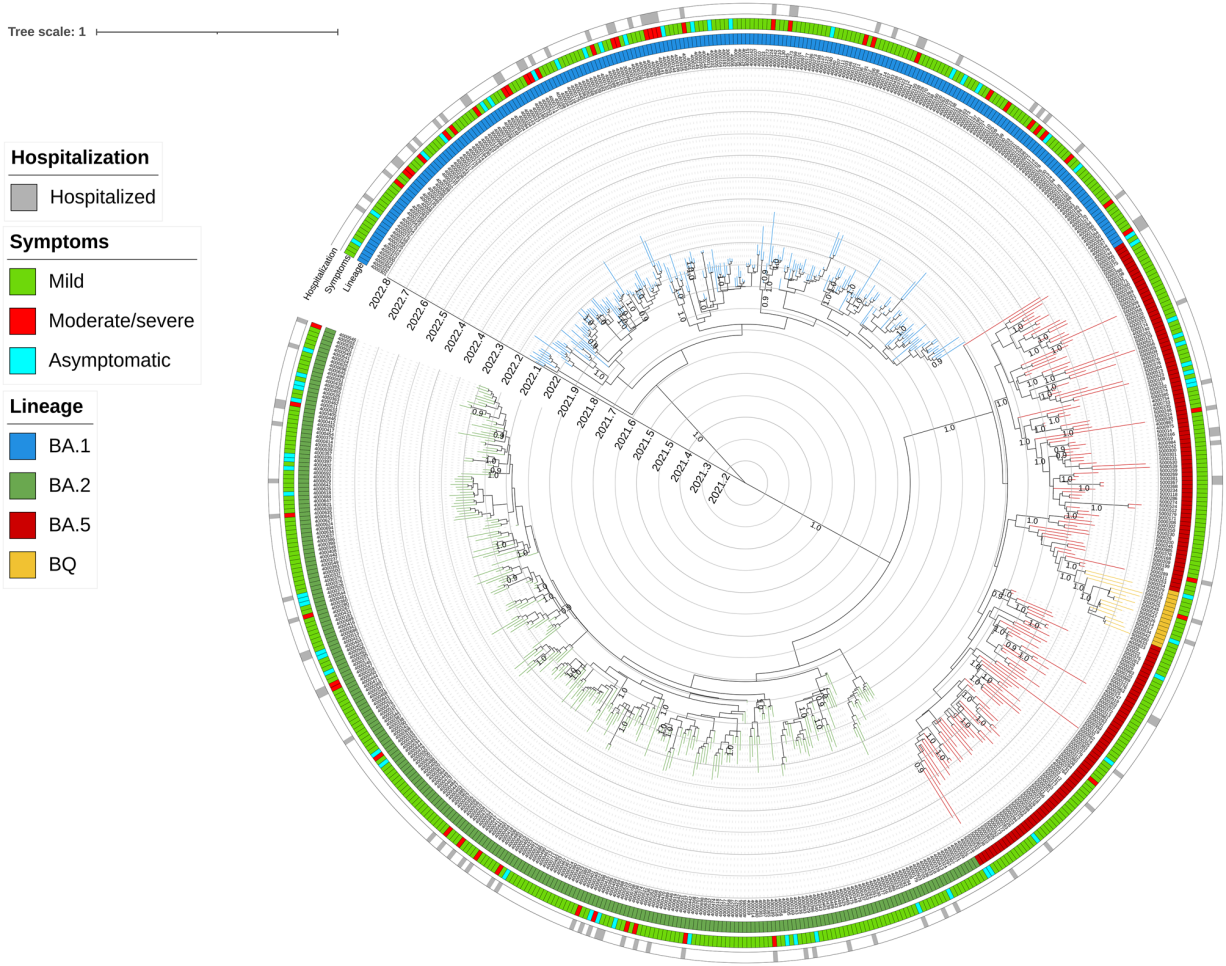
Bayesian phylogenetic reconstruction incorporating date of diagnosis of the 657 SARS-CoV-2 sequences of 29,801 nucleotides of length obtained by population aged ≤ 12 years. SARS-CoV-2 genomes were highlighted in different colors against omicron lineages. Information regarding hospitalization and symptoms were also reported. Three independent chains were run for 50 million states, using the best-fit model of nucleotide substitution GTR + I + G4 with an uncorrelated relaxed molecular clock under a noninformative continuous-time Markov chain (CTMC) reference prior using only paediatric sequences. Parameters and trees were sampled every 1000 states.

The distribution of SARS-CoV-2 sequences against clinical characteristics and against the global context of SARS-CoV-2 (Pangolin https://pangolin.cog-uk.io/)^22^ is shown by the Maximum likelihood tree in Supplementary Fig. S1 and by the time-scale phylogeny in Fig. 1. Demographic and clinical characteristics of patients infected with SARS-CoV-2 against lineages are reported in Table 1.

Whole genome sequencing analysis revealed that at least four major Omicron lineages circulated widely in that period in children. Most of the SARS-CoV-2 infections (n = 266, 40.6%) belonged to the BA.2 lineage, 221 (33.6%) belonged to the BA.1, followed by BA.5 (n = 156, 23.7%) and BQ.1 (n = 14, 2.1%) (Supplementary Fig. S1). A total of 37 sub-lineages were detected. The most prevalent were BA.5.1 (n = 61), BA.2.3 (n = 44) and BA.2.9 (n = 44) followed by BA.5.2 (n = 32), BA.5.2.1 (n = 21). Other 32 sub-lineages were present in less than 20 individuals (Supplementary Data 1).

No many relevant differences in demographic and clinical characteristics were found among Omicron lineages. The only differences regarded age and clinical manifestations. Indeed, most of BA.2 sequences (75.6%) infected children below 1 year of age, followed by BA.5 (n = 104, 66.7%), BQ.1 (n = 9, 64.3%) and BA.1 (n = 135, 61.1%) (p-value: 0.007).

Looking at clinical characteristics, individuals affected by BA.1 lineage showed moderate/severe COVID-19 manifestations (14.9%) more frequently than BA.2 (6.4%), BA.5 (1.9%) and BQ.1 (7.1%) (p-value: < 0.001) (Table 1).

### Evolutionary rate

Evolutionary rates, measured as the number of nucleotide substitutions per site, per year (subs/site/year), were estimated using a Bayesian coalescent method (Fig. 1). The analysis showed a mean Omicron evolutionary rate (subs/site/year) of 9.8 × 10^–4^ (95% HPD, 8.8 × 10^–4^–1.1 × 10^–3^) with modest differences in evolutionary rate among lineages. BA.1 lineage was found to have a mean substitution rate of 1.1 × 10^–3^ (95% HPD, 3.6 × 10^–4^–2.3 × 10^–3^) subs/site/year, BA.2 exhibited a substitution rate of 9.6 × 10^–4^ (95% HPD, 3.3 × 10^–4^–1.9 × 10^–3^) subs/site/year, BA.5 8.3 × 10^–4^ (95% HPD, 3.9 × 10^–4^–1.4 × 10^–3^) subs/site/year while BQ.1 had a 1.1 × 10^–3^ (95% HPD, 3.6 × 10^–4^–2.2 × 10^–3^) subs/site/year.

### Characterization of SNPs

**Figure 2:**
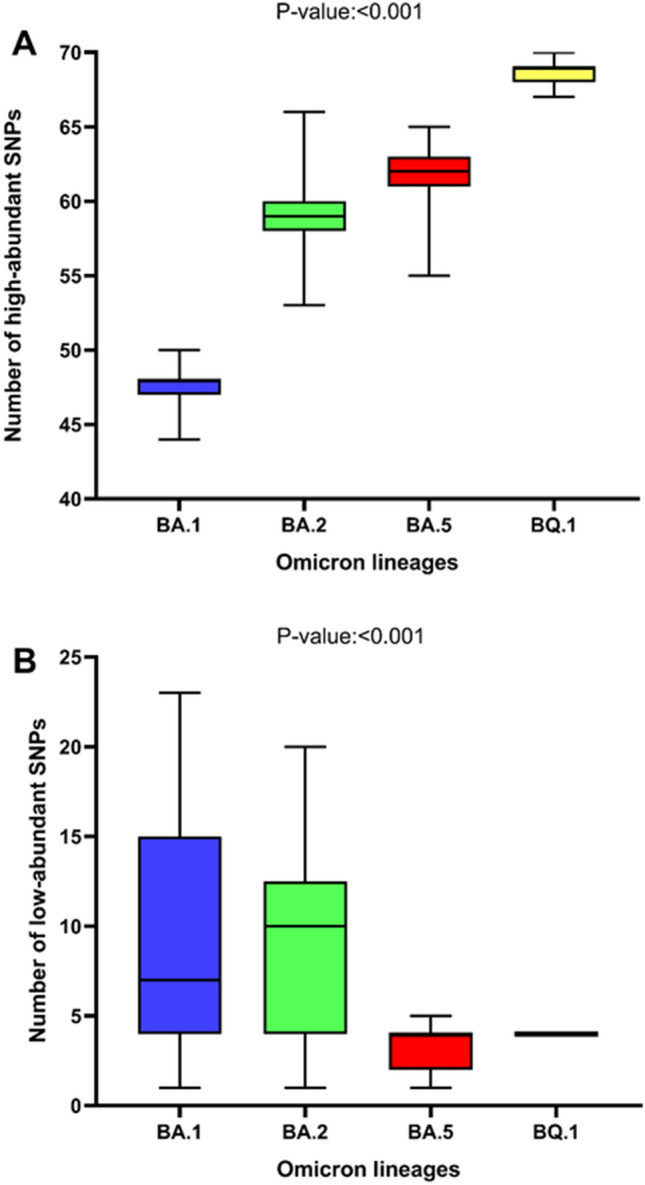
Median number and Interquartile range of high-abundant (**A**) and low-abundant (**B**) SNPs observed against Omicron lineages. p-values were calculated by the Kruskal–Wallis test. *SNPs* single nucleotide polymorphism.

The continuous evolution of the SARS-CoV-2 Omicron clade was well represented by the trend in SNPs prevalence over the lineages. An increasing number of high-abundant SNPs (mutation having a reads frequency ≥ 40%; median [IQR]) was observed among Omicron lineages: 48 (47–48) in BA.1 vs. 59 (58–60) in BA.2 vs. 62 (61–63) in BA.5 vs. 69 (68–69) in BQ.1, p-value: < 0.001 (Fig. 2, Panel A). This increase mainly concerned the spike protein (27 [27–28] in BA.1 vs. 28 [27–28] in BA.2 vs. 29 [29–29] in BA.5 vs. 32 [31–32] in BQ.1, p-value: < 0.001), and non-synonymous SNPs (from 38 [38–40] in BA.1, to 45 [45–46] in BA.2, 48 [47–48] in BA.5, and to 56 [55–56] in BQ.1), thus confirming the highest mutational rate of the spike with respect to the other proteins.

Differently, a significant decrease in the number of low-abundant SNPs (mutation having a reads frequency 2–40%) was observed (BA.1: 7 [4–15] vs. BA.2: 10 [4–12] vs. BA.5: 4 [2–4] vs BQ.1: 4 [4–4], p-value: < 0.001) (Fig. 2, Panel B). This decrease mainly involved non-synonymous SNPs which decreased from 5 (3–13) in BA.1, 8 (3–11) in BA.2 and 3 (3–3) in BA.5 to 3 (3–3) in BQ.1 (p-value: < 0.001).

Supplementary Table S1 reports the 102 SNPs characterized by a different prevalence across Omicron lineages (Supplementary Table S1).

### Covariation profiles among SARS-CoV-2 SNPs

#### Statistically significant pairs of SNPs

Looking at potential associations among SNPs, we found that 69 SNPs were involved in significant associations (Supplementary Table S2). Forty-six resided in non-structural proteins and the remaining 23 in structural ones. Seventeen SNPs were localized in spike positions, 13 in nsp3, and 10 in RNA-dependent RNA polymerase.

Overall, 47 pairs of SNPs showed positive associations (phi > 0.3 and p-value < 0.001) and 47 negative associations (phi <  − 0.3 and p-value < 0.001). The 70.2% (33/47) of positive associations and the 55.3% (n = 26/47) of negative associations involved non-structural proteins and mainly nsp3 and RdRp proteins. The structural protein mostly involved in significant associations was the spike protein, with 16 pairs of SNPs involved in negative association and 9 pairs involved in positive association.

#### Clusters of correlated SNPs

**Figure 3:**
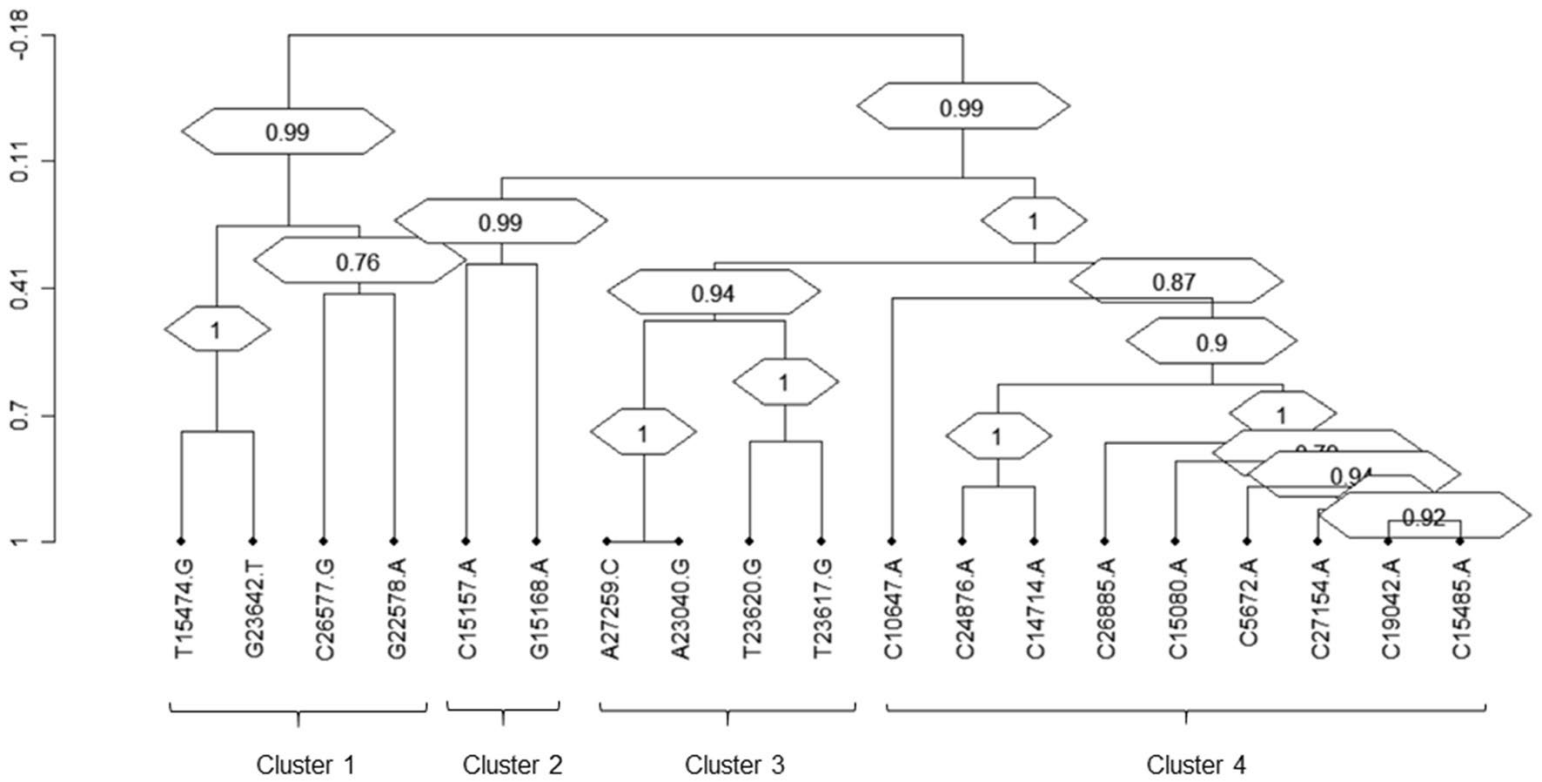
Dendrogram of correlated mutations. The dendrogram, obtained from average linkage hierarchical agglomerative clustering, shows clusters of mutations localized in different SARS-CoV-2 proteins. The length of branches reflects distances between mutations in the original distance matrix. Bootstrap values, indicating the significance of clusters, are reported in the boxes.

By hierarchical clustering analysis, it was possible to identify 4 distinct clusters (bootstrap > 0.75) of SNPs, positively correlated among them (Fig. 3, Table 2).

**Table 2:** Clusters of positively correlated SNPs.

Cluster	SNP 1	Prevalence n (%)	SNP 2	Prevalence n (%)	Covariation freq n (%)^a^	Covariation freq n (%)^b^	Phi^c^	p-value^d^
Cluster 1	G22578A	192 (29.2)	C26577G	460 (70.0)	191 (99.5)	191 (41.5)	0.42	3.05E−36
			G23642T	51 (7.8)	51 (26.6)	51 (100)	0.45	1.16E−29
			T15474G	42 (6.4)	35 (18.2)	35 (100)	0.31	1.88E−13
Cluster 2	G15168A	89 (13.5)	C15157A	57 (8.7)	30 (33.7)	30 (52.6)	0.35	1.91E−13
Cluster 3	T23620G	334 (50.8)	A23040G	487 (74.1)	304 (91.0)	304 (62.4)	0.39	2.64E−24
			A27259C	488 (74.3)	304 (91.0)	304 (62.3)	0.39	5.73E−24
			T23617G	320 (48.7)	288 (43.8)	288 (90.0)	0.76	1.04E−94
Cluster 4	C5672A	184 (28.0)	C27154A	164 (25)	157 (85.3)	157 (95.7)	0.87	1.91E−111
			C26885A	124 (18.9)	120 (65.2)	120 (96.8)	0.74	6.60E−77
			C24876A	89 (13.5)	88 (47.8)	88 (98.9)	0.62	5.56E−55
			C19042A	167 (25.4)	158 (85.9)	158 (94.6)	0.87	3.77E−110
			C15485A	162 (24.7)	156 (84.8)	156 (96.3)	0.87	1.47E−111
			C15080A	130 (19.8)	126 (68.5)	126 (96.9)	0.76	1.95E−82
			C14714A	81 (12.3)	79 (42.9)	79 (97.5)	0.58	9.11E−47
			C10647A	35 (5.3)	35 (19.0)	35 (100)	0.38	1.21E−20

*SNP* single nucleotide polymorphism.

^a^Covariation frequency based on the prevalence of SNP 1.

^b^Covariation frequency based on the prevalence of SNP 2.

^c^Positive correlations with phi > 0.3

^d^All p-values for covariation were significant at a false discovery rate of 0.05.

The first cluster (bootstrap = 0.99) includes 4 SNPs located in the spike protein (G22578A and G23642T), the membrane protein (C26577G) and RdRp (T15474G). Two of these SNPs (G23642T and T15474G) were present at low frequency and low abundance in BA.1, BA.2 and BA.5, while were detected in 100% of BQ.1. C26577G increased its prevalence and frequency among lineages, becoming high-abundant SNP in BA.5 and BQ.1. G22578 in the spike protein was known to be a highly abundant SNP detected in more than 90% of BA.5 and BQ.1 (Supplementary Table S1).

The second cluster (bootstrap = 0.99) involved two low-abundant SNPs (the non-synonymous C15157A and the synonymous G15168A) detected exclusively in the BA.1 and BA.2 lineages and both located in the RdRp region (Supplementary Table S1).

The third cluster (bootstrap = 0.96) is characterized by four SNPs located mainly in the spike and ORF6 gene. Three synonymous SNPs (A27259C, A23040G and T23617G) were exclusively detected in BA.1 and BA.2. The non-synonymous T23620G (never exceeding an abundance of 20%) in the spike protein decreased its prevalence from BA.1 to BA.5, while was detected in the 100% of BQ.1 (Supplementary Table S1).

The fourth large cluster (bootstrap = 0.99) involved 9 low abundant non-synonymous SNPs (C10647A, C1474A, C15080A, C15485A, C19042A, C24876A, C26886A, C27154A and C5672A), located in different regions of the SARS-CoV-2 genome (membrane, nsp3, nsp5, nsp14, RdRp and spike). These SNPs were found only in BA.1 and BA.2 lineages with a prevalence never exceeding 42% (Supplementary Table S1).

### Correlation with moderate/severe COVID-19 manifestation

Univariate and multivariate logistic regression models were performed to define if moderate/severe COVID-19 presentation can potentially be associated with clusters of SNPs, lineages and demographic characteristics (Table 3). As confounding factors, age, gender, and comorbidities were considered. The results showed that in our paediatric population, moderate/severe COVID-19 presentation was negatively associated with patients aged < 1 year (adjusted odds ratio, AOR: 0.40 [0.21–0.79] p-value: 0.007) and positively associated with the third cluster of SNPs (A23040G, A27259C, T23617G, T23620G) (AOR [95% CI] 2.49 [1.26–4.89] p-value: 0.008) and comorbidities (AOR [95% CI] 2.67 [1.36–5.24] p-value: 0.004) (Table 3). A trend of negative association was observed between disease severity and BA.5 (AOR: 0.30 [0.08–1.09] p-value: 0.067).

**Table 3:** Multivariate logistic regression analysis of factors associated with moderate/severe manifestation.

Variable associated to modere/severe	Univariate analysis		Multivariate analysis	
	OR (95% CI)	p-value	AOR (95% CI)	p-value
Gender (male vs. female)	0.89 (0.51–1.55)	0.674		
Age				
< 1	0.28 (0.16–0.50)	< 0.001	0.40 (0.21–0.79)	0.007
1–5	2.84 (1.60–5.04)	< 0.001		0.437
≥ 5	2.17 (1.00–4.68)	0.050		0.401
Comorbidities	4.07 (2.28–7.26)	< 0.001	2.67 (1.36–5.24)	0.004
Omicron lineage				
BA.1	3.47 (1.96–6.16)	< 0.001		0.146
BA.2	0.65 (0.36–1.19)	0.162		
BA.5	0.17 (0.05–0.56)	0.004	0.30 (0.08–1.09)	0.067
BQ.1	0.86 (0.11–6.67)	0.882		
Clusters of SNPs				
Cluster 1 (C26577G + G22578A + G23642T + G28936T + T15474G)	0.36 (0.05–2.70)	0.320		
Cluster 2 (C15157A + G15168A)	1.26 (0.37–4.28)	0.717		
Cluster 3 (A23040G + A27259C + T23617G + T23620G)	3.35 (1.83–6.15)	< 0.001	2.49 (1.26–4.89)	0.008
Cluster 4 (C10647A + C1474A + C15080A + C15485A + C19042A + C24876A + C26886A + C27154A + C5672A)	1.25 (0.28–5.53)	0.769		

*CI* confidence interval, *OR* odds ratio, *AOR* adjusted odds ratio, *RdRp* RNA-dependent RNA polymerase.

## Discussion

The Omicron wave resulted in an increased number of children infected by SARS-CoV-2 even though displaying a reduced incidence of severe manifestations^23,24^ compared to Delta and pre-Delta clades. An in-depth genomic characterization of Omicron variability, taking into consideration both high and low abundance mutations, may address new perspectives on infection and pathogenesis modifications, also in the setting of long-term manifestations and Multisystem inflammatory syndrome (MIS-C), the latter known to be mostly related to young age^25–27^.

Here, our study confirmed the large circulation of SARS-CoV-2 Omicron in the paediatric population, with age less than 1 year appearing to be the most susceptible category to infection regardless of lineage.

Genomic characterization of the whole genome of SARS-CoV-2 confirmed the increase of high-abundant SNPs over time, reaching 69 mutations in BQ.1 lineage, compared to 48 observed in BA.1, with most of the SNPs being localized in the spike protein. As already extensively reported, the accumulation of these mutations defines new lineages known to have increased infectivity, transmission, and immune escape^11,12,28^.

Despite the accumulation of high-abundant SNPs over time, SARS-CoV-2 strains belonging to the different Omicron lineages did not seem to differ consistently in the evolutionary rate from each other, in line with previously published studies^10,29^. The only difference concerned the evolutionary rate of the BQ.1 lineage, which in our case appeared to be slightly higher than that reported in a previous study (1.1 × 10^–3^ [95%HPD, 3.6 × 10^–4^–2.2 × 10^–3^] subs/site/year vs.7.6 × 10^–4^ [95%HPD, 5.2 × 10^–4^–9.8 × 10^–4^] subs/site/year). This slight difference could be due to the few BQ.1 sequences collected in our study-period (n = 14).

Our study also provided evidence of a decrease in low-abundant SNPs from BA.1 to BA.5 and BQ.1 lineages. Similar results were observed by intra-host genetic analysis, which revealed a lower number of mutations in BA.2.3 and BA.5 compared to BA.1 and BA.2 in the spike protein^30^. These results might hypothesize the role of these mutations in immune responses, in line with evidence on the enhanced ability of the omicron clade, particularly the later lineages, to evade the immune system even in vaccinated subjects^31^.

Moreover, a number of these SNPs were involved in mutational clusters together with high abundant and constitutive SNPs. The first complex mutational cluster identified involved the low abundant SNPs RdRp-G678G [nucleotide: T15474G], spike-A694S [nucleotide: G23642T]. These SNPs showed increased prevalence across Omicron lineages. Specifically, their prevalence increased from 10% in the first Omicron lineages to 100% in the BQ.1 lineage, without showing a substantial difference in their reads’ frequency. These SNPs clustered together with the highly abundant SNPs spike-G339D [nucleotide: G22578A] and Membrane-Q19E [nucleotide: C26577G]. The first one, falling in the spike protein RBD domain, can increase the molecular flexibility of the glycoprotein^32^. The second, which resides in the N-terminal domain of the membrane protein, appears to destabilize the structure of the protein itself^33^.

The second cluster involved two low abundant SNPs localized in the RdRp (L576L [nucleotide: G15168A] and Q573K [nucleotide: C15157A]) and exclusively found in BA.1 and BA.2 lineages. Both SNPs reside in the finger domain of RdRp^34^. The close contact of Q573K with the residues involved in the active site and substrate/template binding tunnel as well as with the residues directly involved in RdRp-inhibitors binding might suggest its involvement in replication capacity and drug interaction^35^.

The third cluster, found mainly in BA.1 and BA.2 lineages, was composed of the spike mutations S686R [nucleotide: T23620G], Q493R [nucleotide: A23040G], and R685R [nucleotide: T23617G] together with the ORF6-R20R [nucleotide: A27259C]. The low abundant S686R mutation was detected mainly in BA.1 and BA.2 sequences, and in a minority of BA.5 (10.3% of total BA.5 with S686R), maintaining a reads frequency always below 20%. This mutation is localized close to the furin cleavage site, implicated in the replication and pathogenesis of SARS-CoV-2. A previous study showed that the amino acid change from polar, not charged (serine) to non-polar (glycine) at this position interfered in furin-type cleavages^36^. In our case, the new amino acid (Arginine) is positively charged, and therefore further insights are needed to define the role of this mutation (even if present at low reads frequency) in affecting or improving the recognition of furin cleavage site and in increasing SARS-CoV-2 infectivity. Q493R, located in the receptor binding domain of the spike protein (S1-RBD), increases binding affinity to angiotensin-converting enzyme 2 and reduces susceptibility to class 3 monoclonal antibodies and to bamlanivimab^37,38^. Spike-R685R [nucleotide: T23617G] is localized into furin cleavage site, implicated in replication and pathogenesis of SARS-CoV-2^39^. Finally, ORF6 exhibits critical antagonistic activity, preventing the antiviral innate immune response by inhibiting interferon β (IFN-β) production and blocking the expression of STAT1-activated genes for SARS-CoV-2^40^.

The fourth and last cluster revealed an important participation of non-structural proteins. Specifically, most of the SNPs identified in this cluster are located in proteins essential for viral replication and high replication fidelity, such as major protease (Mpro-Nsp5), papain-like protease (PLpro-Nsp3), exoribonuclease (ExoN-Nsp14), RdRp^41–44^.

Multivariate logistic regression has further sustained these clusters suggesting their predictive role in disease manifestation. Indeed, the multivariate logistic regression model identified cluster 3, mainly found in BA.1 and BA.2 lineages and composed of three spike mutations, as positively associated with the worst clinical manifestation, together with comorbidities. Differently, BA.5 lineage showed a trend of negative association with moderate/severe manifestations. Thus, our multivariate analysis suggests that the ongoing evolution of the Omicron clade is associated with different trend of severe manifestations in children. This is confirmed also when major Omicron lineages were compared with previous circulating Delta clade in paediatric population (AOR [95% CI] 0.22 [0.07–0.72] p-value: 0.012 for BA.5 and 1.73 [0.95–3.14] p-value: 0.074 for Delta clade, data not shown). In line with this, a recent study indicates that Omicron lineages dominating between January and June 2022 caused a less severe disease^45^.

Factors that can contribute to this evidence include increased immunization (both natural and artificial) at the population level and functional properties of the new clade that might impact the pathogenesis of SARS-CoV-2 for humans^46–48^.

In the paediatric setting, these results could also explain the low risk of MIS-C observed after infection by Omicron clade^49^.

Our study has some limitations. Limitations to assessments of the proportions of asymptomatic cases should be noted: most of our paediatric population is tested only when children have symptoms, so relatively few asymptomatic infections are recorded. No information is available regarding the vaccination status. Another limitation is the limited presence of BQ.1 and the absence of the recombined Omicron forms (for example XBB or XBB.1.5), at the time of the study still absent. Since the end of the study (November 2022) to the time of writing (April, 2023) there have been 108 SARS-CoV-2 new diagnoses meeting the criteria defined in “Methods” section (i.e. Ct < 29 or COI > 1000). Considering the estimated prevalence of BQ.1 and XBB forms in these last months^50^, samples belonging to these Omicron forms would be about 60–70.

In conclusion, these findings underscored the widespread circulation of SARS-CoV-2 Omicron variant among children, particularly those under the age of one, even though no notable difference could be identified in their clinical outcomes compared to older age groups. Additionally, the study shed light on low-abundant mutations and their impact on evolutionary processes, suggesting a potential role for viral diversification in influencing disease severity.

## Methods

### Sample collection, and epidemiological data

This retrospective observational study, intended as follow-up of a previous work (Alteri et al., Scientific Report 2022^20^), included 657 SARS-CoV-2-positive nasopharyngeal-swabs, collected from 657 patients aged ≤ 12 years referred for SARS-CoV-2 diagnosis at Bambino Gesù Children Hospital from December 2021 to early November 2022.

Demographics, epidemiological and clinical data were obtained retrospectively by pseudonymized electronic medical records.

As the previous paper^20^, the study protocol was approved by local Research Ethics Committee of Ospedale Pediatrico Bambino Gesù IRCCS (prot. 2384_OPBG_2021), and was conducted under the principles of the 1964 Declaration of Helsinki. Informed consent was waived by the Ethics Committee of Ospedale Pediatrico Bambino Gesù IRCCS following the hospital regulations on observational retrospective studies.

The severity of SARS-CoV-2 infection was defined according to Dong et al., Pediatrics. 2020^51^ and based on the clinical features, laboratory testing, and chest radiograph imaging. Asymptomatic, mild and moderate/severe infections were defined according to Alteri et al., Scientific Report 2022^20^.

### Virus amplification and sequencing

Viral RNAs were extracted from nasopharyngeal swabs by using QIAamp Viral RNA Mini Kit, followed by purification with Agencourt RNAClean XP beads. Both the concentration and the quality of all isolated RNA samples were measured and checked with the Nanodrop.

Amplicons of whole genome sequences of SARS-CoV-2 were generated with a 50 ng viral RNA template, by using CleanPlex SARS-CoV-2 Research and Surveillance Panel, QIAseq DIRECT SARSCoV-2 Kit and Illumina COVIDSeq Assay following manufacters’ protocol. Libraries were then generated using the Nextera DNA Flex library preparation kit with Illumina index adaptors and sequenced on a MiSeq instrument (Illumina, San Diego, CA, USA) with 2 × 150-bp paired-end reads. Raw reads were trimmed for adapters and filtered for quality (Phred score > 28) using Fastp (v0.23.2)^52^. Reference-based assembly was performed with BWA-mem (v0.7.17)^53^ aligning against the GenBank reference genome NC_045512.2 (Wuhan, collection date: December 2019).

SNP variants were called with freebayes (v1.3.2)^54^ and all SNPs having a minimum supporting read frequency of 2% with a depth ≥ 10 were retained.

Synonymous and non-synonymous SNPs characterizing Omicron lineages were defined as high-abundant mutations if characterized by a read frequency ≥ 40%, and low-abundant mutations if characterized by a read frequency between 2 and 40%.

### Phylogenetic analysis and estimation of evolutionary rate

Consensus sequences were generated using the GitHub freely distributed software vcf_consensus_builder^55^ considering all SNPs having a minimum read frequency of 40% (high-abundant mutations). SARS-CoV-2 lineages of the obtained consensus sequences were assigned according to Pangolin application (Pangolin v4.1.1, https://github.com/cov-lineages/pangolin) and then grouped in four major lineages (BA.1, BA.2, BA.5, BQ.1). Sequences were aligned using MAFFT v7.475 and manually inspected using Bioedit. The final alignment comprised 657 sequences of 29,801 nucleotides of length. In order to explore the phylogenetic structure of the paediatric epidemic and evolutionary rate of Omicron clade affecting population aged ≤ 12, a maximum likelihood (ML) phylogeny tree was performed with IqTree2 (v2.1.3)^56^ with 1000 bootstrap replicates, using the best-fit model of nucleotide substitution GTR + F + R3 inferred by ModelFinder^57^. The ML tree was inspected in TempEst^58^, in order to define the correlation between genetic diversity (root-to-tip divergence) and time of sample collection.

Bayesian coalescent methods were further performed, in order to define the phylogenetic structure of the paediatric epidemic against time. A Bayesian coalescent tree analysis was undertaken with BEAST (v1.10.4)^59﻿^, using the GTR + I + G4 substitution model, inferred by Modeltest-NG (v0.1.7)^60,61^, with an exponential population growth tree prior and uncorrelated relaxed molecular clock, under a noninformative continuous-time Markov chain (CTMC) reference prior using only paediatric sequences. Three independent chains were run for 50 million states and parameters and trees were sampled every 1000 states. Upon completion, chains were combined using LogCombiner after removing 10% of states as burn-in and convergence was assessed with Tracer (ESS > 100)^62^. Taxon sets were defined based on SARS-CoV-2 lineages and were used to estimate the time of their most recent common ancestor (tMRCA), as well as the rates of evolutionary change (expressed as nucleotide substitutions per site per year).

Annotation of the phylogenetic tree, including information about lineages, SARS-CoV-2 viral load, symptoms and hospitalization was performed with iTOL (v5)^63﻿^.

### Covariation analysis

The binomial-correlation coefficient (phi) was calculated for each pair of SNPs to assess the strength of co-variation among SNPs. Covariation analysis was conducted including all SARS-CoV-2 SNPs with a prevalence > 5% in the overall population.

The phi coefficient and p-value for all the possible pairwise combinations were calculated by using a script implemented in the R software, version 3.4.1. Statistically significant pairwise associations were considered those with p-value < 0.001 and phi > 0.3 and <  − 0.3.

To analyze the covariation structure of the SNPs in more detail, average linkage hierarchical agglomerative clustering, reported as a dendrogram, was performed. The statistical robustness of the dendrogram was confirmed with a bootstrap analysis using 10.000 replications. Clusters with a bootstrap value equal or higher than 0.70 were considered well-supported.

### Statistical analysis

Descriptive statistics were expressed as median values and interquartile range (IQR) for continuous data and number (percentage) for categorical data. To assess significant differences, chi-squared test for trend and Kruskal–Wallis were used for categorical and continuous variables, respectively.

A multivariate logistic regression analysis was performed to evaluate demographic and virus-related associated with disease severity.

Statistical analyses were performed with SPSS software package for Windows (version 23.0, SPSS Inc., Chicago, IL). A two-sided p-value < 0.05 was considered statistically significant.

### Supplementary Information

Supplementary Information 1.

Supplementary Information 2.

## Data Availability

The SARS-CoV-2 sequences obtained in this study are openly available on European Nucleotide Archive under the Accession Numbers PRJEB63319 (https://www.ebi.ac.uk/ena/browser/view/PRJEB63319). The list of accession numbers and their sublineage is available in the Supplementary Data 1. The de-identified data regarding demographic and clinical features related to each patient are available on reasonable request from the corresponding author. Data are not publicly available to fully comply with the privacy guarantee.
