# Supplementary material for: Molecular characterization of SARS-CoV-2 Omicron clade and clinical presentation in children
Source: Sci Rep. 2024 Mar 4;14:5325. doi: 10.1038/s41598-024-55599-0 (PMC10912656; doi:10.1038/s41598-024-55599-0)
Supplement: Supplementary file 2 — Supplementary Information 2. [file 41598_2024_55599_MOESM2_ESM.zip › SupplTable1_v.2.0.docx]

Supplementary table 1. **Prevalence and reads frequency of 102 SNPs against Omicron lineages.**

| Location | SNP | Type of mutation | Mutation | Prevalence (N, %) | | | | | | Reads frequency (median, IQR) | | | | |
| --- | --- | --- | --- | --- | --- | --- | --- | --- | --- | --- | --- | --- | --- | --- |
|  |  |  |  | Overall | BA.1  N=221 | BA.2  N=266 | BA.5  N=156 | BQ.1  N=14 | P-value | BA.1  N=221 | BA.2  N=266 | BA.5  N=156 | BQ.1  N=14 | P-value |
| Membrane | C26858T | Synonymous | F112F | 265 (40.3) | 0 | 265 (99.6) | 0 | 0 | <0.001 | - | 98.7 (98.1-99.2) | - | - | - |
| Membrane | C26885A | Non-Synonymous | N121K | 124 (18.9) | 48 (21.7) | 76 (28.6) | 0 | 0 | <0.001 | 6.0 (5.6-6.7) | 6.9 (6.1-7.4) | - | - | <0.001 |
| Membrane | A27038G | Synonymous | T172T | 33 (5) | 0 | 0 | 33 (21.2) | 0 | <0.001 | - | - | 100 (100-100) | - | - |
| Membrane | C26577G | Non-Synonymous | Q19E | 461 (70.2) | 151 (68.3) | 141 (53.0) | 155 (99.4) | 14 (100) | <0.001 | 10.9 (7.8-16.1) | 9.6 (7.5-13.9) | 97.7 (90.4-99.4) | 89.9 (87.6-92.8) | <0.001 |
| Membrane | C27154A | Non-Synonymous | S211Y | 164 (25) | 81 (36.7) | 83 (31.2) | 0 | 0 | <0.001 | 10.4 (9.2-11.3) | 7.7 (6.7-8.7) | - | - | <0.001 |
| Membrane | A26530G | Non-Synonymous | D3G | 206 (31.4) | 206 (93.2) | 0 | 0 | 0 | <0.001 | 100 (100-100) | - | - | - | - |
| Membrane | G26529A | Non-Synonymous | D3N | 156 (23.7) | 0 | 0 | 156 (100) | 14 (100) | <0.001 | - | - | 100 (99.9-100) | 100 (100-100) | 0.321 |
| Nucleocapside | G28936T | Non-Synonymous | L221F | 95 (14.5) | 23 (10.4) | 43 (16.2) | 15 (9.6) | 14 (100) | <0.001 | 5.4 (5.2-6.1) | 6.2 (5.4-7.4) | 6.2 (6-6.6) | 6.1 (5.8-6.5) | 0.013 |
| Nucleocapside | A29510C | Non-Synonymous | S413R | 436 (66.4) | 0 | 266 (200) | 156 (100) | 14 (100) | <0.001 | - | 99.9 (99.8-99.9) | 100 (99.9-100) | 100 (99.9-100) | <0.001 |
| nsp2 | C1627T | Synonymous | L274L | 72 (11) | 0 | 0 | 72 (46.2) | 0 | <0.001 | - | - | 86.4 (82.3-92) | - | - |
| nsp2 | C1902A | Non-Synonymous | S366* | 35 (5.3) | 34 (15.4) | 1 (0.4) | 0 | 0 | <0.001 | 5.3 (5.1-5.5) | 5.2 | - | - | 0.620 |
| nsp2 | C2470T | Synonymous | A555A | 95 (14.5) | 95 (43.0) | 0 | 0 | 0 | <0.001 | 99.7 (99.5-99.9) | - | - | - | - |
| nsp3 | G5924A | Non-Synonymous | V1069I | 50 (7.6) | 50 (22.6) | 0 | 0 | 0 | <0.001 | 91.9 (89.5-93.9) | - | - | - | - |
| nsp3 | T6074A | Non-Synonymous | F1119I | 50 (7.6) | 4 (1.8) | 46 (17.3) | 0 | 0 | <0.001 | 5.6 (5.4-6.1) | 5.8 (5.3-6.7) | - | - | 0.915 |
| nsp3 | T7904A | Non-Synonymous | S1729T | 38 (5.8) | 5 (2.3) | 33 (12.4) | 0 | 0 | <0.001 | 5.3 (5.2-5.4) | 5.6 (5.3-6.3) | - | - | 0.066 |
| nsp3 | C3241T | Synonymous | D174D | 36 (5.5) | 35 (15.8) | 1 (0.4) | 0 | 0 | <0.001 | 99.8 (98.8-99.9) | 12.2 | - | - | 0.111 |
| nsp3 | T8166A | Non-Synonymous | I1816N | 36 (5.5) | 5 (2.3) | 31 (11.7) | 0 | 0 | <0.001 | 6.3 (5.2-6.5) | 5.8 (5.3-7) | - | - | 0.784 |
| nsp3 | G8393A | Non-Synonymous | A1892T | 221 (33.6) | 221 (100) | 0 | 0 | 0 | <0.001 | 83.8 (80.9-87) | - | - | - | - |
| nsp3 | T8523A | Non-Synonymous | V1935D | 40 (6.1) | 5 (2.3) | 35 (13.9) | 0 | 0 | <0.001 | 6.3 (6.2-7) | 5.4 (5.2-6) | - | - | 0.205 |
| nsp3 | C2790T | Non-Synonymous | T24I | 436 (66.4) | 0 | 266 (100) | 156 (100) | 14 (100) | <0.001 | - | 99.6 (99.2-99.7) | 100 (99.9-100) | 100 (100-100) | <0.001 |
| nsp3 | C3634A | Non-Synonymous | N305K | 79 (12) | 55 (24.9) | 24 (9.0) | 0 | 0 | <0.001 | 5.6 (5.4-6.3) | 5.4 (5.3-5.7) | - | - | 0.034 |
| nsp3 | A2832G | Non-Synonymous | K38R | 221 (33.6) | 221 (100) | 0 | 0 | 0 | <0.001 | 99.9 (99.8-100) | - | - | - | - |
| nsp3 | G4184A | Non-Synonymous | G489S | 436 (66.4) | 0 | 266 (100) | 156 (100) | 14 (100) | <0.001 | - | 99.9 (99.8-100) | 100 (99.9-100) | 100 (99.9-100) | <0.001 |
| nsp3 | C4321T | Synonymous | A534A | 436 (66.4) | 0 | 266 (100) | 156 (100) | 14 (100) | <0.001 | - | 93.3 (90.9-94.7) | 99.4 (97.9-99.7) | 98.9 (98.6-99.3) | <0.001 |
| nsp3 | G4953A | Non-Synonymous | R745K | 47 (7.2) | 4 (1.8) | 43 (16.2) | 0 | 0 | <0.001 | 5.5 (5.3-5.6) | 6 (5.4-6.3) | - | - | 0.230 |
| nsp3 | C5140A | Non-Synonymous | D807E | 44 (6.7) | 42 (19) | 2 (0.8) | 0 | 0 | <0.001 | 6 (5.6-6.6) | 6 (5.2-6.7) | - | - | 0.757 |
| nsp3 | T5386G | Synonymous | A889A | 221 (33.6) | 221 (100) | 0 | 0 | 0 | <0.001 | 98.6 (97.1-99.4) | - | - | - | - |
| nsp3 | G5529A | Non-Synonymous | C937Y | 39 (5.9) | 1 (0.5) | 38 (14.3) | 0 | 0 | <0.001 | 6.5 | 5.5 (5.3-5.8) | - | - | 0.110 |
| nsp3 | C5672A | Non-Synonymous | P985T | 184 (28) | 92 (41.6) | 92 (34.6) | 0 | 0 | <0.001 | 12.4 (9.7-13.8) | 8.7 (7.5-10.4) | - | - | <0.001 |
| nsp4 | C8991T | Non-Synonymous | A146V | 49 (7.5) | 0 | 49 (18.4) | 0 | 0 | <0.001 | - | 99.9 (99.8-99.9) | - | - | - |
| nsp4 | C9344T | Non-Synonymous | L264F | 436 (66.4) | 0 | 266 (100) | 156 (100) | 14 (100) | <0.001 | - | 99.9 (99.8-100) | 100 (99.9-100) | 100 (99.9-100) | <0.001 |
| nsp4 | A9424G | Synonymous | V290V | 315 (47.9) | 0 | 146 (54.9) | 155 (99.4) | 14 (100) | <0.001 | - | 100 (100-100) | 99.9 (99.9-100) | 100 (99.9-100) | <0.001 |
| nsp4 | C9534T | Non-Synonymous | T327I | 436 (66.4) | 0 | 266 (100) | 156 (100) | 14 (100) | <0.001 | - | 98.7 (98.3-99.1) | 100 (99.9-100) | 100 (100-100) | <0.001 |
| nsp4 | C9866T | Non-Synonymous | L438F | 265 (40.3) | 0 | 265 (99.6) | 0 | 0 | <0.001 | - | 99.9 (99.8-100) | - | - | - |
| nsp5 | G10447A | Synonymous | R131R | 436 (66.4) | 0 | 266 (100) | 156 (100) | 14 (100) | <0.001 | - | 99.4 (99.2-99.6) | 99.8 (99.7-99.9) | 99.7 (99.6-99.7) | <0.001 |
| nsp5 | C10647A | Non-Synonymous | T198K | 35 (5.3) | 32 (14.5) | 3 (1.1) | 0 | 0 | <0.001 | 5.4 (5.1-5.7) | 5.8 (5.2-8.6) | - | - | 0.263 |
| nsp5 | C10198T | Synonymous | D48D | 427 (65) | 0 | 257 (96.6) | 156 (100) | 14 (100) | <0.001 | - | 100 (100-100) | 99.9 (99.8-100) | 99.9 (99.8-100) | <0.001 |
| nsp5 | T10234A | Synonymous | R60R | 41 (6.2) | 4 (1.8) | 37 (13.9) | 0 | 0 | <0.001 | 5.9 (5.7-7) | 6 (5.6-7.5) | - | - | 0.93 |
| nsp6 | A11537G | Non-Synonymous | I189V | 221 (33.6) | 221 (100) | 0 | 0 | 0 | <0.001 | 99.9 (99.8-100) | - | - | - | - |
| nsp6 | T11709A | Non-Synonymous | V246D | 45 (6.8) | 3 (1.4) | 42 (15.8) | 0 | 0 | <0.001 | 5.3 (5.1-5.6) | 5.7 (5.3-6.3) | - | - | 0.202 |
| nsp7 | C11871A | Non-Synonymous | S10* | 48 (7.3) | 37 (16.7) | 11 (4.1) | 0 | 0 | <0.001 | 5.7 (5.2-6.1) | 5.3 (5.2-5.6) | - | - | 0.215 |
| nsp8 | G12160A | Synonymous | E23E | 170 (25.9) | 0 | 0 | 156 (100) | 14 (100) | <0.001 | - | - | 99.9 (99.9-100) | 100 (99.9-100) | 0.001 |
| nsp8 | G12310A | Synonymous | Q73Q | 35 (5.3) | 0 | 0 | 35 (22.4) | 0 | <0.001 | - | - | 99.9 (99.9-100) | - | - |
| nsp9 | C12880T | Synonymous | I65I | 403 (61.3) | 0 | 233 (87.6) | 156 (100) | 14 (100) | <0.001 | - | 100 (100-100) | 100 (99.9-100) | 100 (99.9-100) | <0.001 |
| nsp10 | T13195C | Synonymous | V57V | 221 (33.6) | 221 (100) | 0 | 0 | 0 | <0.001 | 99.9 (99.8-100) | - | - | - | - |
| nsp13 | T16548A | Synonymous | T104T | 40 (6.1) | 4 (1.8) | 36 (13.5) | 0 | 0 | <0.001 | 6.3 (6.1-7) | 6.3 (5.5-7.1) | - | - | 0.588 |
| nsp13 | C17410T | Non-Synonymous | R392C | 436 (66.4) | 0 | 266 (100) | 156 (100) | 14 (100) | <0.001 | - | 70.4 (67.1-75) | 100 (99.9-100) | 100 (100-100) | <0.001 |
| nsp14 | C19042A | Non-Synonymous | P335T | 167 (25.4) | 80 (36.2) | 87 (32.7) | 0 | 0 | <0.001 | 9.3 (8.3-10.7) | 7.8 (6.7-8.6) | - | - | <0.001 |
| nsp14 | T19427A | Non-Synonymous | I463K | 44 (6.7) | 5 (2.3) | 39 (14.7) | 0 | 0 | <0.001 | 5.1 (5-5.9) | 5.8 (5.2-6.1) | - | - | 0.385 |
| nsp14 | C18060T | Non-Synonymous | L7L | 39 (5.9) | 0 | 39 (14.7) | 0 | 0 | <0.001 | - | 99.9 (99.7-100) | - | - | - |
| nsp14 | G18315A | Synonymous | E92E | 38 (5.8) | 0 | 38 (14.3) | 0 | 0 | <0.001 | - | 99.7 (99.6-99.9) | - | - | - |
| nsp15 | C19955T | Non-Synonymous | T112I | 436 (66.4) | 0 | 266 (100) | 156 (100) | 14 (100) | <0.001 | - | 100 (99.9-100) | 100 (99.9-100) | 100 (100-100) | 0.826 |
| nsp15 | A20055G | Synonymous | E145E | 436 (66.4) | 0 | 266 (100) | 156 (100) | 14 (100) | <0.001 | - | 100 (99.8-100) | 99.9 (99.9-100) | 100 (99.9-100) | 0.350 |
| nsp15 | G20111A | Non-Synonymous | G164E | 42 (6.4) | 3 (1.4) | 39 (14.7) | 0 | 0 | <0.001 | 5.2 (5.1-5.3) | 5.9 (5.4-6.5) | - | - | 0.010 |
| nsp15 | G20263A | Non-Synonymous | E215K | 41 (6.2) | 3 (1.4) | 38 (14.3) | 0 | 0 | <0.001 | 5.8 (5.3-6.1) | 5.8 (5.4-6.4) | - | - | 0.764 |
| nsp15 | T20529A | Synonymous | V303V | 44 (6.7) | 2 (0.9) | 42 (15.8) | 0 | 0 | <0.001 | 5.7 (5.1-6.3) | 5.8 (5.3-6.5) | - | - | 0.693 |
| ORF3a | C25810T | Non-Synonymous | L140F | 48 (7.3) | 0 | 48 (18) | 0 | 0 | <0.001 | - | 99.9 (99.7-100) | - | - | - |
| ORF3a | C26060T | Non-Synonymous | T223I | 436 (66.4) | 0 | 266 (100) | 156 (100) | 14 (100) | <0.001 | - | 99.8 (99.5-99.9) | 100 (99.9-100) | 100 (100-100) | <0.001 |
| ORF3a | C25624T | Non-Synonymous | H78Y | 41 (6.2) | 0 | 41(15.4) | 0 | 0 | <0.001 | - | 99.8 (99.7-100) | - | - | - |
| ORF6 | A27259C | Synonymous | R20R | 488 (74.3) | 221 (100) | 266 (100) | 1 (0.6) | 0 | <0.001 | 99.5 (99-99.8) | 99.4 (98.5-99.8) | 17.5 | - | 0.009 |
| ORF6 | G27382C | Non-Synonymous | D61L | 265 (40.3) | 0 | 265 (99.6) | 0 | 0 | <0.001 | - | 98.5 (97.9-99.2) | - | - | - |
| ORF7a | T27438C | Synonymous | C15C | 69 (10.5) | 0 | 0 | 69 (44.2) | 0 | <0.001 | - | - | 99.9 (99.8-100) | - | - |
| RdRp | C14292A | Non-Synonymous | D284E | 72 (11) | 52 (23.5) | 20(7.5) | 0 | 0 | <0.001 | 5.9 (5.5-6.8) | 5.5 (5.2-5.8) | - | - | 0.005 |
| RdRp | T14537A | Non-Synonymous | L366H | 39 (5.9) | 2 (0.9) | 37 (13.9) | 0 | 0 | <0.001 | 6 (5.7-6.4) | 5.5 (5.3-6.2) | - | - | 0.339 |
| RdRp | C14714A | Non-Synonymous | S425Y | 81 (12.3) | 73 (33) | 8 (3.0) | 0 | 0 | <0.001 | 6.8 (6.1-7.4) | 5.4 (5.2-6.3) | - | - | 0.007 |
| RdRp | C15080A | Non-Synonymous | A547D | 130 (19.8) | 75 (33.9) | 55 (20.7) | 0 | 0 | <0.001 | 7.4 (6.4-8.4) | 6.1 (5.5-7.3) | - | - | <0.001 |
| RdRp | C15157A | Non-Synonymous | Q573K | 57 (8.7) | 42 (19) | 15 (5.6) | 0 | 0 | <0.001 | 6.1 (5.7-6.6) | 6.2 (6-7.1) | - | - | 0.201 |
| RdRp | G15168A | Synonymous | L576L | 89 (13.5) | 80 (36.2) | 9 (3.4) | 0 | 0 | <0.001 | 6.3 (5.8-7) | 6.1 (5.7-6.2) | - | - | 0.149 |
| RdRp | C15173A | Non-Synonymous | S578* | 42 (6.4) | 37 (16.7) | 4 (1.5) | 1 (0.6) | 0 | <0.001 | 6.2 (5.7-7.1) | 6.1 (6-7.2) | 6.3 | - | 0.900 |
| RdRp | C15240T | Synonymous | N600N | 221 (33.6) | 221 (100) | 0 | 0 | 0 | <0.001 | 99.9 (99.8-100) | - | - | - | - |
| RdRp | T15474G | Synonymous | G678G | 42 (6.4) | 8 (3.6) | 6 (2.3) | 14 (9.0) | 14 (100) | <0.001 | 5.9 (5.4-6.2) | 5.3 (5.1-5.7) | 6.4 (6.0-7.0) | 6.5 (6.3-7.3) | 0.002 |
| RdRp | C15485A | Non-Synonymous | S682* | 162 (24.7) | 80 (36.2) | 82 (30.8) | 0 | 0 | <0.001 | 11 (9.7-12) | 7.6 (7-8.9) | - | - | <0.001 |
| RdRp | C15714T | Synonymous | L758L | 436 (66.4) | 0 | 266 (100) | 156 (100) | 14 (100) | <0.001 | - | 99.9 (99.7-100) | 99.9 (99.9-100) | 100 (100-100) | 0.002 |
| Spike | C24876A | Non-Synonymous | T1105K | 89 (13.5) | 72 (32.6) | 17 (6.4) | 0 | 0 | <0.001 | 7 (6.1-7.5) | 5.7 (5.4-6.4) | - | - | <0.001 |
| Spike | G21987A | Non-Synonymous | G142D | 433 (65.9) | 0 | 266 (100) | 155 (99.4) | 12 (85.7) | <0.001 | - | 94.1 (92.7-95.2) | 99.9 (99.8-100) | 100 (99.9-100) | <0.001 |
| Spike | C21618T | Non-Synonymous | T19I | 436 (66.4) | 0 | 266 (100) | 156 (100) | 14 (100) | <0.001 | - | 100 (99.9-100) | 100 (99.9-100) | 100 (99.9-100) | 0.352 |
| Spike | T22200G | Non-Synonymous | V213G | 435 (66.2) | 0 | 265 (99.6) | 156 (100) | 14 (100) | <0.001 | - | 98.8 (96.5-99.6) | 99.9 (99.8-99.9) | 100 (99.9-100) | <0.001 |
| Spike | T22207G | Non-Synonymous | D215E | 37 (5.6) | 0 | 37 (13.9) | 0 | 0 | <0.001 | - | 99.6 (99-100) | - | - | - |
| Spike | G22578A | Non-Synonymous | G339D | 192 (29.2) | 10 (4.5) | 14 (5.3) | 154 (98.7) | 14 (100) | <0.0010 | 100 (100-100) | 99.9 (99.9-100) | 100 (99.9-100) | 100 (99.9-100) | 0.038 |
| Spike | C22674T | Non-Synonymous | S371F | 430 (65.4) | 0 | 260 (97.7) | 156 (100) | 14 (100) | <0.001 | - | 100 (99.8-100) | 99.7 (99.5-99.9) | 99.9 (99.7-99.9) | <0.001 |
| Spike | T22673C | Non-Synonymous | S371L | 214 (32.6) | 214 (96.8) | 0 | 0 | 0 | <0.001 | 100 (100-100) | - | - | - | - |
| Spike | A22688G | Non-Synonymous | T376A | 433 (65.9) | 0 | 263 (98.9) | 156 (100) | 14 (100) | <0.001 | - | 99.9 (99.6-100) | 99.9 (99.8-100) | 99.9 (99.9-100) | 0.312 |
| Spike | G22775A | Non-Synonymous | D405N | 436 (66.4) | 0 | 266 (100) | 156 (100) | 14 (100) | <0.001 | - | 76.9 (73.8-79.8) | 100 (99.9-100) | 100 (100-100) | <0.001 |
| Spike | A22786C | Non-Synonymous | R408S | 435 (66.2) | 0 | 266 (100) | 155 (99.4) | 14 (100) | <0.001 | - | 99.9 (99.8-100) | 82.9 (79.7-86.5) | 82.6 (79.7-83.8) | <0.001 |
| Spike | C22792T | Synonymous | I410I | 113 (17.2) | 0 | 112 (42.1) | 1 (0.6) | 0 | <0.001 | - | 99.9 (99.8-100) | 98.8 | - | 0.106 |
| Spike | G22898A | Non-Synonymous | G446S | 220 (33.5) | 220 (99.5) | 0 | 0 | 0 | <0.001 | 99.9 (99.8-100) | - | - | - | - |
| Spike | T22917G | Non-Synonymous | L452R | 164 (25) | 0 | 0 | 150 (96.2) | 14 (100) | <0.001 | - | - | 100 (99.9-100) | 100 (100-100) | 0.216 |
| Spike | T23018G | Non-Synonymous | F486V | 170 (25.9) | 0 | 0 | 156 (100) | 14 (100) | <0.001 | - | - | 100 (99.9-100) | 100 (100-100) | 0.265 |
| Spike | A23040G | Non-Synonymous | Q493R | 487 (74.1) | 221 (100) | 266 (100) | 0 | 0 | <0.001 | 100 (99.5-100) | 100 (99.9-100) | - | - | <0.001 |
| Spike | G23048A | Non-Synonymous | G496S | 221 (33.6) | 221 (100) | 0 | 0 | 0 | <0.001 | 100 (99.8-100) | - | - | - | - |
| Spike | C23202A | Non-Synonymous | T547K | 221 (33.6) | 221 (100) | 0 | 0 | 0 | <0.001 | 99.8 (99.6-100) | - | - | - | - |
| Spike | A23614C | Synonymous | A684A | 63 (9.6) | 44 (19.9) | 19 (7.1) | 0 | 0 | <0.001 | 6 (5.4-7.1) | 6.8 (5.5-8.1) | - | - | 0.330 |
| Spike | C23613G | Non-Synonymous | A684G | 147 (22.4) | 82 (37.1) | 65 (24.4) | 0 | 0 | <0.001 | 8.3 (6.7-9.5) | 6.3 (5.8-7.8) | - | - | <0.001 |
| Spike | T23617G | Synonymous | R685R | 320 (48.7) | 193 (87.3) | 127 (47.7) | 0 | 0 | <0.001 | 10.9 (9.5-12) | 9.9 (8.7-11.1) | - | - | <0.001 |
| Spike | T23620G | Non-Synonymous | S686R | 334 (50.8) | 182 (82.4) | 122 (45.9) | 16 (10.3) | 14 (100) | <0.001 | 12 (10.6-13.3) | 14.1 (11.6-15.9) | 9.4 (8.8-10.0) | 10.1 (9.3-11.2) | <0.001 |
| Spike | T23622G | Non-Synonymous | V687G | 50 (7.6) | 33 (14.9) | 17 (6.4) | 0 | 0 | <0.001 | 9.4 (8.2-11.0) | 9.1 (8.2-11.1) | - | - | 0.79 |
| Spike | G23642T | Non-Synonymous | A694S | 51 (7.8) | 8 (3.6) | 13 (4.9) | 16 (10.3) | 14 (100) | <0.001 | 11.7 (10.4-12.3) | 12.5 (11.8-13.3) | 8.4 (7.9-8.9) | 7.8 (7.6-8.8) | <0.001 |
| Spike | C23664T | Non-Synonymous | A701V | 39 (5.9) | 39 (17.6) | 0 | 0 | 0 | <0.001 | 99.8 (99.5-100) | - | - | - | - |
| Spike | C23700A | Non-Synonymous | A713D | 97 (14.8) | 28 (12.7) | 69 (25.9) | 0 | 0 | <0.001 | 7.7 (6-9.4) | 7.4 (6.1-9.2) | - | - | 0.714 |
| Spike | C24130A | Non-Synonymous | N856K | 221 (33.6) | 221 (100) | 0 | 0 | 0 | <0.001 | 99.3 (98.9-99.6) | - | - | - | - |
| Spike | C21846T | Non-Synonymous | T95I | 221 (33.6) | 221 (100) | 0 | 0 | 0 | <0.001 | 82.6 (79.8-86.4) | - | - | - | - |
| Spike | C24503T | Non-Synonymous | L981F | 221 (33.6) | 221 (100) | 0 | 0 | 0 | <0.001 | 99.7 (99.5-99.8) | - | - | - | - |

Prevalence and reads frequency are expressed as N (%) and median (Interquartile range), respectively. Two-sided P-values were calculated by Mann-whitney, Kruskal–Wallis test, or Chi-square test for trend, as appropriate. RdRp: RNA-dependent RNA polymerase; Nsp: non-structural protein.
