# Supplementary material for: Molecular characterization of SARS-CoV-2 Omicron clade and clinical presentation in children
Source: Sci Rep. 2024 Mar 4;14:5325. doi: 10.1038/s41598-024-55599-0 (PMC10912656; doi:10.1038/s41598-024-55599-0)
Supplement: Supplementary file 2 — Supplementary Information 2. [file 41598_2024_55599_MOESM2_ESM.zip › SupplTable2_2.0.docx]

Supplementary table 2: **Positively and negatively correlated pairs of SNPs**

| SNP 1 | Prevalence,  n (%) | SNP 2 | Prevalence,  n (%) | Covariation frequency,  n (%)^a^ | Covariation frequency,  n (%)^b^ | Phi | P-value^c^ |
| --- | --- | --- | --- | --- | --- | --- | --- |
| G22578A | 192 (29.2) | T23617G | 320 (48.7) | 1 (0.5) | 1 (0.3) | -0.619462614 | 1.86709E-69 |
| C12880T | 403 (61.3) | T23617G | 320 (48.7) | 98 (24.3) | 98 (30.6) | -0.614608226 | 1.8609E-59 |
| A9424G | 315 (47.9) | T23617G | 320 (48.7) | 54 (17.1) | 54 (16.9) | -0.60603988 | 6.30259E-57 |
| G12160A | 170 (25.9) | T23617G | 320 (48.7) | 0 (0) | 0 (0) | -0.575731536 | 2.17649E-61 |
| G26529A | 170 (25.9) | T23617G | 320 (48.7) | 0 (0) | 0 (0) | -0.575731536 | 2.17649E-61 |
| T23018G | 170 (25.9) | T23617G | 320 (48.7) | 0 (0) | 0 (0) | -0.575731536 | 2.17649E-61 |
| T22917G | 164 (25) | T23617G | 320 (48.7) | 0 (0) | 0 (0) | -0.562028713 | 1.38105E-58 |
| C10198T | 427 (65) | T23617G | 320 (48.7) | 120 (28.1) | 120 (37.5) | -0.561642603 | 1.05405E-49 |
| A20055G | 436 (66.4) | T23617G | 320 (48.7) | 127 (29.1) | 127 (39.7) | -0.550156727 | 1.31754E-47 |
| A29510C | 436 (66.4) | T23617G | 320 (48.7) | 127 (29.1) | 127 (39.7) | -0.550156727 | 1.31754E-47 |
| C15714T | 436 (66.4) | T23617G | 320 (48.7) | 127 (29.1) | 127 (39.7) | -0.550156727 | 1.31754E-47 |
| C17410T | 436 (66.4) | T23617G | 320 (48.7) | 127 (29.1) | 127 (39.7) | -0.550156727 | 1.31754E-47 |
| C19955T | 436 (66.4) | T23617G | 320 (48.7) | 127 (29.1) | 127 (39.7) | -0.550156727 | 1.31754E-47 |
| C21618T | 436 (66.4) | T23617G | 320 (48.7) | 127 (29.1) | 127 (39.7) | -0.550156727 | 1.31754E-47 |
| C26060T | 436 (66.4) | T23617G | 320 (48.7) | 127 (29.1) | 127 (39.7) | -0.550156727 | 1.31754E-47 |
| C2790T | 436 (66.4) | T23617G | 320 (48.7) | 127 (29.1) | 127 (39.7) | -0.550156727 | 1.31754E-47 |
| C4321T | 436 (66.4) | T23617G | 320 (48.7) | 127 (29.1) | 127 (39.7) | -0.550156727 | 1.31754E-47 |
| C9344T | 436 (66.4) | T23617G | 320 (48.7) | 127 (29.1) | 127 (39.7) | -0.550156727 | 1.31754E-47 |
| C9534T | 436 (66.4) | T23617G | 320 (48.7) | 127 (29.1) | 127 (39.7) | -0.550156727 | 1.31754E-47 |
| G10447A | 436 (66.4) | T23617G | 320 (48.7) | 127 (29.1) | 127 (39.7) | -0.550156727 | 1.31754E-47 |
| G22775A | 436 (66.4) | T23617G | 320 (48.7) | 127 (29.1) | 127 (39.7) | -0.550156727 | 1.31754E-47 |
| G4184A | 436 (66.4) | T23617G | 320 (48.7) | 127 (29.1) | 127 (39.7) | -0.550156727 | 1.31754E-47 |
| A22786C | 435 (66.2) | T23617G | 320 (48.7) | 127 (29.2) | 127 (39.7) | -0.546411081 | 7.61101E-47 |
| T22200G | 435 (66.2) | T23617G | 320 (48.7) | 127 (29.2) | 127 (39.7) | -0.546411081 | 7.61101E-47 |
| A22688G | 433 (65.9) | T23617G | 320 (48.7) | 126 (29.1) | 126 (39.4) | -0.545387326 | 7.6082E-47 |
| C22674T | 430 (65.4) | T23617G | 320 (48.7) | 124 (28.8) | 124 (38.8) | -0.540704788 | 6.73374E-46 |
| G21987A | 433 (65.9) | T23617G | 320 (48.7) | 127 (29.3) | 127 (39.7) | -0.538963297 | 1.13099E-45 |
| C12880T | 403 (61.3) | T23620G | 334 (50.8) | 122 (30.3) | 122 (36.5) | -0.518129185 | 7.0153E-42 |
| A9424G | 315 (47.9) | T23620G | 334 (50.8) | 82 (26) | 82 (24.6) | -0.476188828 | 1.03502E-34 |
| A20055G | 436 (66.4) | G15168A | 89 (13.5) | 9 (2.1) | 9 (10.1) | -0.471268623 | 3.6311E-32 |
| A29510C | 436 (66.4) | G15168A | 89 (13.5) | 9 (2.1) | 9 (10.1) | -0.471268623 | 3.6311E-32 |
| C15714T | 436 (66.4) | G15168A | 89 (13.5) | 9 (2.1) | 9 (10.1) | -0.471268623 | 3.6311E-32 |
| C17410T | 436 (66.4) | G15168A | 89 (13.5) | 9 (2.1) | 9 (10.1) | -0.471268623 | 3.6311E-32 |
| C19955T | 436 (66.4) | G15168A | 89 (13.5) | 9 (2.1) | 9 (10.1) | -0.471268623 | 3.6311E-32 |
| C21618T | 436 (66.4) | G15168A | 89 (13.5) | 9 (2.1) | 9 (10.1) | -0.471268623 | 3.6311E-32 |
| C26060T | 436 (66.4) | G15168A | 89 (13.5) | 9 (2.1) | 9 (10.1) | -0.471268623 | 3.6311E-32 |
| C2790T | 436 (66.4) | G15168A | 89 (13.5) | 9 (2.1) | 9 (10.1) | -0.471268623 | 3.6311E-32 |
| C4321T | 436 (66.4) | G15168A | 89 (13.5) | 9 (2.1) | 9 (10.1) | -0.471268623 | 3.6311E-32 |
| C9344T | 436 (66.4) | G15168A | 89 (13.5) | 9 (2.1) | 9 (10.1) | -0.471268623 | 3.6311E-32 |
| C9534T | 436 (66.4) | G15168A | 89 (13.5) | 9 (2.1) | 9 (10.1) | -0.471268623 | 3.6311E-32 |
| G10447A | 436 (66.4) | G15168A | 89 (13.5) | 9 (2.1) | 9 (10.1) | -0.471268623 | 3.6311E-32 |
| G22775A | 436 (66.4) | G15168A | 89 (13.5) | 9 (2.1) | 9 (10.1) | -0.471268623 | 3.6311E-32 |
| G4184A | 436 (66.4) | G15168A | 89 (13.5) | 9 (2.1) | 9 (10.1) | -0.471268623 | 3.6311E-32 |
| A22786C | 435 (66.2) | G15168A | 89 (13.5) | 9 (2.1) | 9 (10.1) | -0.46947237 | 5.53822E-32 |
| T22200G | 435 (66.2) | G15168A | 89 (13.5) | 9 (2.1) | 9 (10.1) | -0.46947237 | 5.53822E-32 |
| C10198T | 427 (65) | T23620G | 334 (50.8) | 144 (33.7) | 144 (43.1) | -0.466422353 | 5.35869E-34 |
| A22688G | 433 (65.9) | G15168A | 89 (13.5) | 9 (2.1) | 9 (10.1) | -0.465907906 | 1.28284E-31 |
| G21987A | 433 (65.9) | G15168A | 89 (13.5) | 9 (2.1) | 9 (10.1) | -0.465907906 | 1.28284E-31 |
| C10198T | 427 (65) | G15168A | 89 (13.5) | 8 (1.9) | 8 (9) | -0.464755452 | 6.05964E-32 |
| C22674T | 430 (65.4) | G15168A | 89 (13.5) | 9 (2.1) | 9 (10.1) | -0.460629684 | 4.33771E-31 |
| G21987A | 433 (65.9) | T23620G | 334 (50.8) | 150 (34.6) | 150 (44.9) | -0.450396092 | 1.06745E-31 |
| A20055G | 436 (66.4) | T23620G | 334 (50.8) | 152 (34.9) | 152 (45.5) | -0.448819936 | 1.65324E-31 |
| A29510C | 436 (66.4) | T23620G | 334 (50.8) | 152 (34.9) | 152 (45.5) | -0.448819936 | 1.65324E-31 |
| C15714T | 436 (66.4) | T23620G | 334 (50.8) | 152 (34.9) | 152 (45.5) | -0.448819936 | 1.65324E-31 |
| C17410T | 436 (66.4) | T23620G | 334 (50.8) | 152 (34.9) | 152 (45.5) | -0.448819936 | 1.65324E-31 |
| C19955T | 436 (66.4) | T23620G | 334 (50.8) | 152 (34.9) | 152 (45.5) | -0.448819936 | 1.65324E-31 |
| C21618T | 436 (66.4) | T23620G | 334 (50.8) | 152 (34.9) | 152 (45.5) | -0.448819936 | 1.65324E-31 |
| C26060T | 436 (66.4) | T23620G | 334 (50.8) | 152 (34.9) | 152 (45.5) | -0.448819936 | 1.65324E-31 |
| C2790T | 436 (66.4) | T23620G | 334 (50.8) | 152 (34.9) | 152 (45.5) | -0.448819936 | 1.65324E-31 |
| C4321T | 436 (66.4) | T23620G | 334 (50.8) | 152 (34.9) | 152 (45.5) | -0.448819936 | 1.65324E-31 |
| C9344T | 436 (66.4) | T23620G | 334 (50.8) | 152 (34.9) | 152 (45.5) | -0.448819936 | 1.65324E-31 |
| C9534T | 436 (66.4) | T23620G | 334 (50.8) | 152 (34.9) | 152 (45.5) | -0.448819936 | 1.65324E-31 |
| G10447A | 436 (66.4) | T23620G | 334 (50.8) | 152 (34.9) | 152 (45.5) | -0.448819936 | 1.65324E-31 |
| G22775A | 436 (66.4) | T23620G | 334 (50.8) | 152 (34.9) | 152 (45.5) | -0.448819936 | 1.65324E-31 |
| G4184A | 436 (66.4) | T23620G | 334 (50.8) | 152 (34.9) | 152 (45.5) | -0.448819936 | 1.65324E-31 |
| A20055G | 436 (66.4) | C14714A | 81 (12.3) | 8 (1.8) | 8 (9.9) | -0.44832798 | 3.41038E-29 |
| A29510C | 436 (66.4) | C14714A | 81 (12.3) | 8 (1.8) | 8 (9.9) | -0.44832798 | 3.41038E-29 |
| C15714T | 436 (66.4) | C14714A | 81 (12.3) | 8 (1.8) | 8 (9.9) | -0.44832798 | 3.41038E-29 |
| C17410T | 436 (66.4) | C14714A | 81 (12.3) | 8 (1.8) | 8 (9.9) | -0.44832798 | 3.41038E-29 |
| C19955T | 436 (66.4) | C14714A | 81 (12.3) | 8 (1.8) | 8 (9.9) | -0.44832798 | 3.41038E-29 |
| C21618T | 436 (66.4) | C14714A | 81 (12.3) | 8 (1.8) | 8 (9.9) | -0.44832798 | 3.41038E-29 |
| C26060T | 436 (66.4) | C14714A | 81 (12.3) | 8 (1.8) | 8 (9.9) | -0.44832798 | 3.41038E-29 |
| C2790T | 436 (66.4) | C14714A | 81 (12.3) | 8 (1.8) | 8 (9.9) | -0.44832798 | 3.41038E-29 |
| C4321T | 436 (66.4) | C14714A | 81 (12.3) | 8 (1.8) | 8 (9.9) | -0.44832798 | 3.41038E-29 |
| C9344T | 436 (66.4) | C14714A | 81 (12.3) | 8 (1.8) | 8 (9.9) | -0.44832798 | 3.41038E-29 |
| C9534T | 436 (66.4) | C14714A | 81 (12.3) | 8 (1.8) | 8 (9.9) | -0.44832798 | 3.41038E-29 |
| G10447A | 436 (66.4) | C14714A | 81 (12.3) | 8 (1.8) | 8 (9.9) | -0.44832798 | 3.41038E-29 |
| G22775A | 436 (66.4) | C14714A | 81 (12.3) | 8 (1.8) | 8 (9.9) | -0.44832798 | 3.41038E-29 |
| G4184A | 436 (66.4) | C14714A | 81 (12.3) | 8 (1.8) | 8 (9.9) | -0.44832798 | 3.41038E-29 |
| A22786C | 435 (66.2) | C14714A | 81 (12.3) | 8 (1.8) | 8 (9.9) | -0.446624225 | 4.95873E-29 |
| T22200G | 435 (66.2) | C14714A | 81 (12.3) | 8 (1.8) | 8 (9.9) | -0.446624225 | 4.95873E-29 |
| A22786C | 435 (66.2) | T23620G | 334 (50.8) | 152 (34.9) | 152 (45.5) | -0.445050083 | 5.85844E-31 |
| T22200G | 435 (66.2) | T23620G | 334 (50.8) | 152 (34.9) | 152 (45.5) | -0.445050083 | 5.85844E-31 |
| A22688G | 433 (65.9) | T23620G | 334 (50.8) | 151 (34.9) | 151 (45.2) | -0.443973314 | 8.69293E-31 |
| A22688G | 433 (65.9) | C14714A | 81 (12.3) | 8 (1.8) | 8 (9.9) | -0.443243358 | 1.0455E-28 |
| G21987A | 433 (65.9) | C14714A | 81 (12.3) | 8 (1.8) | 8 (9.9) | -0.443243358 | 1.0455E-28 |
| C10198T | 427 (65) | C14714A | 81 (12.3) | 7 (1.6) | 7 (8.6) | -0.443012413 | 3.88817E-29 |
| C22674T | 430 (65.4) | T23620G | 334 (50.8) | 150 (34.9) | 150 (44.9) | -0.439203616 | 4.35261E-30 |
| C22674T | 430 (65.4) | C14714A | 81 (12.3) | 8 (1.9) | 8 (9.9) | -0.43823712 | 3.17461E-28 |
| C12880T | 403 (61.3) | G15168A | 89 (13.5) | 7 (1.7) | 7 (7.9) | -0.434671885 | 2.69873E-29 |
| C12880T | 403 (61.3) | C24876A | 89 (13.5) | 9 (2.2) | 9 (10.1) | -0.416405323 | 1.22154E-26 |
| C12880T | 403 (61.3) | C14714A | 81 (12.3) | 6 (1.5) | 6 (7.4) | -0.415311442 | 6.71496E-27 |
| C23700A | 97 (14.8) | C26577G | 461 (70.2) | 25 (25.8) | 25 (5.4) | -0.403836222 | 2.33221E-22 |
| G22578A | 192 (29.2) | C5672A | 184 (28) | 0 (0) | 0 (0) | -0.400776713 | 1.45624E-33 |
| A20055G | 436 (66.4) | C24876A | 89 (13.5) | 17 (3.9) | 17 (19.1) | -0.395959636 | 1.87116E-22 |
| A29510C | 436 (66.4) | C24876A | 89 (13.5) | 17 (3.9) | 17 (19.1) | -0.395959636 | 1.87116E-22 |
| C15714T | 436 (66.4) | C24876A | 89 (13.5) | 17 (3.9) | 17 (19.1) | -0.395959636 | 1.87116E-22 |
| C17410T | 436 (66.4) | C24876A | 89 (13.5) | 16 (3.7) | 16 (18) | -0.395959636 | 1.87116E-22 |
| C19955T | 436 (66.4) | C24876A | 89 (13.5) | 17 (3.9) | 17 (19.1) | -0.395959636 | 1.87116E-22 |
| C21618T | 436 (66.4) | C24876A | 89 (13.5) | 17 (3.9) | 17 (19.1) | -0.395959636 | 1.87116E-22 |
| C26060T | 436 (66.4) | C24876A | 89 (13.5) | 17 (3.9) | 17 (19.1) | -0.395959636 | 1.87116E-22 |
| C2790T | 436 (66.4) | C24876A | 89 (13.5) | 17 (3.9) | 17 (19.1) | -0.395959636 | 1.87116E-22 |
| C4321T | 436 (66.4) | C24876A | 89 (13.5) | 17 (3.9) | 17 (19.1) | -0.395959636 | 1.87116E-22 |
| C9344T | 436 (66.4) | C24876A | 89 (13.5) | 17 (3.9) | 17 (19.1) | -0.395959636 | 1.87116E-22 |
| C9534T | 436 (66.4) | C24876A | 89 (13.5) | 17 (3.9) | 17 (19.1) | -0.395959636 | 1.87116E-22 |
| G10447A | 436 (66.4) | C24876A | 89 (13.5) | 17 (3.9) | 17 (19.1) | -0.395959636 | 1.87116E-22 |
| G22775A | 436 (66.4) | C24876A | 89 (13.5) | 17 (3.9) | 17 (19.1) | -0.395959636 | 1.87116E-22 |
| G4184A | 436 (66.4) | C24876A | 89 (13.5) | 17 (3.9) | 17 (19.1) | -0.395959636 | 1.87116E-22 |
| A22786C | 435 (66.2) | C24876A | 89 (13.5) | 17 (3.9) | 17 (19.1) | -0.394246872 | 2.65633E-22 |
| T22200G | 435 (66.2) | C24876A | 89 (13.5) | 17 (3.9) | 17 (19.1) | -0.394246872 | 2.65633E-22 |
| G12160A | 170 (25.9) | T23620G | 334 (50.8) | 30 (17.6) | 30 (9) | -0.392246566 | 2.64636E-24 |
| G26529A | 170 (25.9) | T23620G | 334 (50.8) | 30 (17.6) | 30 (9) | -0.392246566 | 2.64636E-24 |
| T23018G | 170 (25.9) | T23620G | 334 (50.8) | 30 (17.6) | 30 (9) | -0.392246566 | 2.64636E-24 |
| A22688G | 433 (65.9) | C24876A | 89 (13.5) | 17 (3.9) | 17 (19.1) | -0.390846235 | 5.2965E-22 |
| G21987A | 433 (65.9) | C24876A | 89 (13.5) | 17 (3.9) | 17 (19.1) | -0.390846235 | 5.2965E-22 |
| C22674T | 430 (65.4) | C24876A | 89 (13.5) | 17 (4) | 17 (19.1) | -0.38580601 | 1.47499E-21 |
| C10198T | 427 (65) | C24876A | 89 (13.5) | 17 (4) | 17 (19.1) | -0.380836345 | 4.04593E-21 |
| A23040G | 487 (74.1) | C26577G | 461 (70.2) | 293 (60.2) | 293 (63.6) | -0.377649254 | 4.40018E-29 |
| A27259C | 488 (74.3) | C26577G | 461 (70.2) | 293 (60) | 293 (63.6) | -0.376106119 | 4.72163E-29 |
| T22917G | 164 (25) | T23620G | 334 (50.8) | 30 (18.3) | 30 (9) | -0.375462312 | 2.47952E-22 |
| G22578A | 192 (29.2) | C19042A | 167 (25.4) | 0 (0) | 0 (0) | -0.375132191 | 6.64167E-30 |
| G22578A | 192 (29.2) | T23620G | 334 (50.8) | 42 (21.9) | 42 (12.6) | -0.372259371 | 1.57704E-21 |
| G22578A | 192 (29.2) | C27154A | 164 (25) | 0 (0) | 0 (0) | -0.370614667 | 3.06219E-29 |
| G12160A | 170 (25.9) | C5672A | 184 (28) | 0 (0) | 0 (0) | -0.368500703 | 7.12543E-29 |
| G26529A | 170 (25.9) | C5672A | 184 (28) | 0 (0) | 0 (0) | -0.368500703 | 7.12543E-29 |
| T23018G | 170 (25.9) | C5672A | 184 (28) | 0 (0) | 0 (0) | -0.368500703 | 7.12543E-29 |
| G22578A | 192 (29.2) | C15485A | 162 (24.7) | 0 (0) | 0 (0) | -0.367602999 | 1.38313E-28 |
| T22917G | 164 (25) | C5672A | 184 (28) | 0 (0) | 0 (0) | -0.359730122 | 8.88907E-28 |
| A9424G | 315 (47.9) | C5672A | 184 (28) | 36 (11.4) | 36 (19.6) | -0.35431341 | 4.41286E-20 |
| A9424G | 315 (47.9) | C24876A | 89 (13.5) | 3 (1) | 3 (3.4) | -0.35318607 | 1.0569E-22 |
| A20055G | 436 (66.4) | C5140A | 44 (6.7) | 2 (0.5) | 2 (4.5) | -0.350532746 | 1.23734E-18 |
| A29510C | 436 (66.4) | C5140A | 44 (6.7) | 2 (0.5) | 2 (4.5) | -0.350532746 | 1.23734E-18 |
| C15714T | 436 (66.4) | C5140A | 44 (6.7) | 2 (0.5) | 2 (4.5) | -0.350532746 | 1.23734E-18 |
| C17410T | 436 (66.4) | C5140A | 44 (6.7) | 2 (0.5) | 2 (4.5) | -0.350532746 | 1.23734E-18 |
| C19955T | 436 (66.4) | C5140A | 44 (6.7) | 2 (0.5) | 2 (4.5) | -0.350532746 | 1.23734E-18 |
| C21618T | 436 (66.4) | C5140A | 44 (6.7) | 2 (0.5) | 2 (4.5) | -0.350532746 | 1.23734E-18 |
| C26060T | 436 (66.4) | C5140A | 44 (6.7) | 2 (0.5) | 2 (4.5) | -0.350532746 | 1.23734E-18 |
| C2790T | 436 (66.4) | C5140A | 44 (6.7) | 2 (0.5) | 2 (4.5) | -0.350532746 | 1.23734E-18 |
| C4321T | 436 (66.4) | C5140A | 44 (6.7) | 2 (0.5) | 2 (4.5) | -0.350532746 | 1.23734E-18 |
| C9344T | 436 (66.4) | C5140A | 44 (6.7) | 2 (0.5) | 2 (4.5) | -0.350532746 | 1.23734E-18 |
| C9534T | 436 (66.4) | C5140A | 44 (6.7) | 2 (0.5) | 2 (4.5) | -0.350532746 | 1.23734E-18 |
| G10447A | 436 (66.4) | C5140A | 44 (6.7) | 2 (0.5) | 2 (4.5) | -0.350532746 | 1.23734E-18 |
| G22775A | 436 (66.4) | C5140A | 44 (6.7) | 2 (0.5) | 2 (4.5) | -0.350532746 | 1.23734E-18 |
| G4184A | 436 (66.4) | C5140A | 44 (6.7) | 2 (0.5) | 2 (4.5) | -0.350532746 | 1.23734E-18 |
| A22786C | 435 (66.2) | C5140A | 44 (6.7) | 2 (0.5) | 2 (4.5) | -0.349282005 | 1.51557E-18 |
| T22200G | 435 (66.2) | C5140A | 44 (6.7) | 2 (0.5) | 2 (4.5) | -0.349282005 | 1.51557E-18 |
| A22688G | 433 (65.9) | C5140A | 44 (6.7) | 2 (0.5) | 2 (4.5) | -0.346800822 | 2.27079E-18 |
| G21987A | 433 (65.9) | C5140A | 44 (6.7) | 2 (0.5) | 2 (4.5) | -0.346800822 | 2.27079E-18 |
| G12160A | 170 (25.9) | C19042A | 167 (25.4) | 0 (0) | 0 (0) | -0.344921428 | 9.91546E-26 |
| G26529A | 170 (25.9) | C19042A | 167 (25.4) | 0 (0) | 0 (0) | -0.344921428 | 9.91546E-26 |
| T23018G | 170 (25.9) | C19042A | 167 (25.4) | 0 (0) | 0 (0) | -0.344921428 | 9.91546E-26 |
| C22674T | 430 (65.4) | C5140A | 44 (6.7) | 2 (0.5) | 2 (4.5) | -0.343128637 | 4.13136E-18 |
| A9424G | 315 (47.9) | C14714A | 81 (12.3) | 2 (0.6) | 2 (2.5) | -0.3413589 | 1.76432E-21 |
| G12160A | 170 (25.9) | C27154A | 164 (25) | 0 (0) | 0 (0) | -0.340767717 | 4.54019E-25 |
| G26529A | 170 (25.9) | C27154A | 164 (25) | 0 (0) | 0 (0) | -0.340767717 | 4.54019E-25 |
| T23018G | 170 (25.9) | C27154A | 164 (25) | 0 (0) | 0 (0) | -0.340767717 | 4.54019E-25 |
| C10198T | 427 (65) | C5140A | 44 (6.7) | 2 (0.5) | 2 (4.5) | -0.339514167 | 7.47142E-18 |
| G12160A | 170 (25.9) | C15485A | 162 (24.7) | 0 (0) | 0 (0) | -0.337998589 | 9.62087E-25 |
| G26529A | 170 (25.9) | C15485A | 162 (24.7) | 0 (0) | 0 (0) | -0.337998589 | 9.62087E-25 |
| T23018G | 170 (25.9) | C15485A | 162 (24.7) | 0 (0) | 0 (0) | -0.337998589 | 9.62087E-25 |
| G22578A | 192 (29.2) | C23613G | 147 (22.4) | 1 (0.5) | 1 (0.7) | -0.336952482 | 1.00724E-23 |
| T22917G | 164 (25) | C19042A | 167 (25.4) | 0 (0) | 0 (0) | -0.336712051 | 1.04818E-24 |
| T22917G | 164 (25) | C27154A | 164 (25) | 0 (0) | 0 (0) | -0.332657201 | 4.48481E-24 |
| T22917G | 164 (25) | C15485A | 162 (24.7) | 0 (0) | 0 (0) | -0.329953981 | 9.59236E-24 |
| A9424G | 315 (47.9) | C19042A | 167 (25.4) | 33 (10.5) | 33 (19.8) | -0.329360156 | 2.12526E-17 |
| A9424G | 315 (47.9) | G15168A | 89 (13.5) | 6 (1.9) | 6 (6.7) | -0.326477593 | 7.87691E-19 |
| A9424G | 315 (47.9) | C23613G | 147 (22.4) | 26 (8.3) | 26 (17.7) | -0.325171678 | 3.44882E-17 |
| A9424G | 315 (47.9) | C27154A | 164 (25) | 33 (10.5) | 33 (20.1) | -0.321220629 | 1.49696E-16 |
| C12880T | 403 (61.3) | C5672A | 184 (28) | 67 (16.6) | 67 (36.4) | -0.319251913 | 1.86006E-15 |
| G22578A | 192 (29.2) | C15080A | 130 (19.8) | 0 (0) | 0 (0) | -0.319146809 | 3.63039E-22 |
| A20055G | 436 (66.4) | C1902A | 35 (5.3) | 2 (0.5) | 2 (5.7) | -0.318840506 | 9.94509E-16 |
| A29510C | 436 (66.4) | C1902A | 35 (5.3) | 1 (0.2) | 1 (2.9) | -0.318840506 | 9.94509E-16 |
| C15714T | 436 (66.4) | C1902A | 35 (5.3) | 1 (0.2) | 1 (2.9) | -0.318840506 | 9.94509E-16 |
| C17410T | 436 (66.4) | C1902A | 35 (5.3) | 1 (0.2) | 1 (2.9) | -0.318840506 | 9.94509E-16 |
| C19955T | 436 (66.4) | C1902A | 35 (5.3) | 1 (0.2) | 1 (2.9) | -0.318840506 | 9.94509E-16 |
| C21618T | 436 (66.4) | C1902A | 35 (5.3) | 1 (0.2) | 1 (2.9) | -0.318840506 | 9.94509E-16 |
| C26060T | 436 (66.4) | C1902A | 35 (5.3) | 1 (0.2) | 1 (2.9) | -0.318840506 | 9.94509E-16 |
| C2790T | 436 (66.4) | C1902A | 35 (5.3) | 1 (0.2) | 1 (2.9) | -0.318840506 | 9.94509E-16 |
| C4321T | 436 (66.4) | C1902A | 35 (5.3) | 1 (0.2) | 1 (2.9) | -0.318840506 | 9.94509E-16 |
| C9344T | 436 (66.4) | C1902A | 35 (5.3) | 1 (0.2) | 1 (2.9) | -0.318840506 | 9.94509E-16 |
| C9534T | 436 (66.4) | C1902A | 35 (5.3) | 1 (0.2) | 1 (2.9) | -0.318840506 | 9.94509E-16 |
| G10447A | 436 (66.4) | C1902A | 35 (5.3) | 1 (0.2) | 1 (2.9) | -0.318840506 | 9.94509E-16 |
| G22775A | 436 (66.4) | C1902A | 35 (5.3) | 1 (0.2) | 1 (2.9) | -0.318840506 | 9.94509E-16 |
| G4184A | 436 (66.4) | C1902A | 35 (5.3) | 1 (0.2) | 1 (2.9) | -0.318840506 | 9.94509E-16 |
| A22786C | 435 (66.2) | C1902A | 35 (5.3) | 1 (0.2) | 1 (2.9) | -0.317723694 | 1.16911E-15 |
| T22200G | 435 (66.2) | C1902A | 35 (5.3) | 1 (0.2) | 1 (2.9) | -0.317723694 | 1.16911E-15 |
| G12160A | 170 (25.9) | C23613G | 147 (22.4) | 0 (0) | 0 (0) | -0.317200278 | 3.53232E-22 |
| G26529A | 170 (25.9) | C23613G | 147 (22.4) | 0 (0) | 0 (0) | -0.317200278 | 3.53232E-22 |
| T23018G | 170 (25.9) | C23613G | 147 (22.4) | 0 (0) | 0 (0) | -0.317200278 | 3.53232E-22 |
| C12880T | 403 (61.3) | C23613G | 147 (22.4) | 48 (11.9) | 48 (32.7) | -0.316260547 | 4.15718E-15 |
| A9424G | 315 (47.9) | C15485A | 162 (24.7) | 33 (10.5) | 33 (20.4) | -0.315765641 | 5.33005E-16 |
| A22688G | 433 (65.9) | C1902A | 35 (5.3) | 1 (0.2) | 1 (2.9) | -0.315508398 | 1.61268E-15 |
| G21987A | 433 (65.9) | C1902A | 35 (5.3) | 1 (0.2) | 1 (2.9) | -0.315508398 | 1.61268E-15 |
| C12880T | 403 (61.3) | C5140A | 44 (6.7) | 2 (0.5) | 2 (4.5) | -0.312459904 | 6.17075E-16 |
| C22674T | 430 (65.4) | C1902A | 35 (5.3) | 1 (0.2) | 1 (2.9) | -0.312230234 | 2.60671E-15 |
| C12880T | 403 (61.3) | C3634A | 79 (12) | 16 (4) | 16 (20.3) | -0.311919261 | 9.81609E-15 |
| C12880T | 403 (61.3) | C15080A | 130 (19.8) | 40 (9.9) | 40 (30.8) | -0.311788797 | 1.04939E-14 |
| G22578A | 192 (29.2) | C26885A | 124 (18.9) | 0 (0) | 0 (0) | -0.309935538 | 6.16761E-21 |
| T22917G | 164 (25) | C23613G | 147 (22.4) | 0 (0) | 0 (0) | -0.309650684 | 3.25728E-21 |
| C10198T | 427 (65) | C1902A | 35 (5.3) | 1 (0.2) | 1 (2.9) | -0.309004192 | 4.17702E-15 |
| A20055G | 436 (66.4) | C15173A | 42 (6.4) | 5 (1.1) | 5 (11.9) | -0.301210913 | 1.51243E-13 |
| A29510C | 436 (66.4) | C15173A | 42 (6.4) | 5 (1.1) | 5 (11.9) | -0.301210913 | 1.51243E-13 |
| C15714T | 436 (66.4) | C15173A | 42 (6.4) | 5 (1.1) | 5 (11.9) | -0.301210913 | 1.51243E-13 |
| C17410T | 436 (66.4) | C15173A | 42 (6.4) | 5 (1.1) | 5 (11.9) | -0.301210913 | 1.51243E-13 |
| C19955T | 436 (66.4) | C15173A | 42 (6.4) | 5 (1.1) | 5 (11.9) | -0.301210913 | 1.51243E-13 |
| C21618T | 436 (66.4) | C15173A | 42 (6.4) | 4 (0.9) | 4 (9.5) | -0.301210913 | 1.51243E-13 |
| C26060T | 436 (66.4) | C15173A | 42 (6.4) | 5 (1.1) | 5 (11.9) | -0.301210913 | 1.51243E-13 |
| C2790T | 436 (66.4) | C15173A | 42 (6.4) | 5 (1.1) | 5 (11.9) | -0.301210913 | 1.51243E-13 |
| C4321T | 436 (66.4) | C15173A | 42 (6.4) | 5 (1.1) | 5 (11.9) | -0.301210913 | 1.51243E-13 |
| C9344T | 436 (66.4) | C15173A | 42 (6.4) | 5 (1.1) | 5 (11.9) | -0.301210913 | 1.51243E-13 |
| C9534T | 436 (66.4) | C15173A | 42 (6.4) | 5 (1.1) | 5 (11.9) | -0.301210913 | 1.51243E-13 |
| G10447A | 436 (66.4) | C15173A | 42 (6.4) | 5 (1.1) | 5 (11.9) | -0.301210913 | 1.51243E-13 |
| G22775A | 436 (66.4) | C15173A | 42 (6.4) | 5 (1.1) | 5 (11.9) | -0.301210913 | 1.51243E-13 |
| G4184A | 436 (66.4) | C15173A | 42 (6.4) | 5 (1.1) | 5 (11.9) | -0.301210913 | 1.51243E-13 |
| A22786C | 435 (66.2) | C15173A | 42 (6.4) | 5 (1.1) | 5 (11.9) | -0.300036043 | 1.78743E-13 |
| T22200G | 435 (66.2) | C15173A | 42 (6.4) | 5 (1.1) | 5 (11.9) | -0.300036043 | 1.78743E-13 |
| T8523A | 40 (6.1) | T8166A | 36 (5.5) | 13 (32.5) | 13 (36.1) | 0.30230817 | 3.79261E-08 |
| C3241T | 36 (5.5) | C5140A | 44 (6.7) | 14 (38.9) | 14 (31.8) | 0.310069641 | 1.22267E-08 |
| G22578A | 192 (29.2) | T15474G | 42 (6.4) | 35 (18.2) | 35 (83.3) | 0.310920675 | 1.87995E-13 |
| T23620G | 334 (50.8) | C14714A | 81 (12.3) | 75 (22.5) | 75 (92.6) | 0.313209808 | 3.0574E-17 |
| A27259C | 488 (74.3) | C23613G | 147 (22.4) | 147 (30.1) | 147 (100) | 0.315941751 | 7.53673E-22 |
| A23040G | 487 (74.1) | C23613G | 147 (22.4) | 147 (30.2) | 147 (100) | 0.317200278 | 3.53232E-22 |
| G28936T | 95 (14.5) | T23620G | 334 (50.8) | 85 (89.5) | 85 (25.4) | 0.317747989 | 4.23723E-17 |
| T11709A | 45 (6.8) | T7904A | 38 (5.8) | 15 (33.3) | 15 (39.5) | 0.320016225 | 3.34473E-09 |
| C11871A | 48 (7.3) | C10647A | 35 (5.3) | 15 (31.3) | 15 (42.9) | 0.324062876 | 2.3942E-09 |
| G20263A | 41 (6.2) | T16548A | 40 (6.1) | 15 (36.6) | 15 (37.5) | 0.329043038 | 1.7581E-09 |
| G15168A | 89 (13.5) | T23617G | 320 (48.7) | 81 (91) | 81 (25.3) | 0.335033256 | 6.0808E-19 |
| A27259C | 488 (74.3) | C15485A | 162 (24.7) | 162 (33.2) | 162 (100) | 0.336657543 | 1.05211E-24 |
| A23040G | 487 (74.1) | C15485A | 162 (24.7) | 162 (33.3) | 162 (100) | 0.337998589 | 9.62087E-25 |
| A27259C | 488 (74.3) | C27154A | 164 (25) | 164 (33.6) | 164 (100) | 0.339415683 | 4.75801E-25 |
| G5529A | 39 (5.9) | T16548A | 40 (6.1) | 15 (38.5) | 15 (37.5) | 0.340108332 | 7.84351E-10 |
| A23040G | 487 (74.1) | C27154A | 164 (25) | 164 (33.7) | 164 (100) | 0.340767717 | 4.54019E-25 |
| T8166A | 36 (5.5) | T7904A | 38 (5.8) | 14 (38.9) | 14 (36.8) | 0.341450371 | 1.33379E-09 |
| T23620G | 334 (50.8) | C26885A | 124 (18.9) | 107 (32) | 107 (86.3) | 0.342052964 | 2.5269E-19 |
| C23613G | 147 (22.4) | G15168A | 89 (13.5) | 52 (35.4) | 52 (58.4) | 0.342435106 | 3.03392E-15 |
| A27259C | 488 (74.3) | C19042A | 167 (25.4) | 167 (34.2) | 167 (100) | 0.343552914 | 2.16565E-25 |
| T23620G | 334 (50.8) | C24876A | 89 (13.5) | 84 (25.1) | 84 (94.4) | 0.344785385 | 9.15789E-21 |
| A23040G | 487 (74.1) | C19042A | 167 (25.4) | 167 (34.3) | 167 (100) | 0.344921428 | 9.91546E-26 |
| T8523A | 40 (6.1) | T7904A | 38 (5.8) | 15 (37.5) | 15 (39.5) | 0.345936115 | 5.04859E-10 |
| T23617G | 320 (48.7) | C14292A | 72 (11) | 71 (22.2) | 71 (98.6) | 0.350273014 | 1.87116E-22 |
| C14292A | 72 (11) | C10647A | 35 (5.3) | 20 (27.8) | 20 (57.1) | 0.3507124 | 1.06279E-11 |
| C14292A | 72 (11) | C1902A | 35 (5.3) | 20 (27.8) | 20 (57.1) | 0.3507124 | 1.06279E-11 |
| G15168A | 89 (13.5) | C15157A | 57 (8.7) | 30 (33.7) | 30 (52.6) | 0.352022035 | 1.91418E-13 |
| T10234A | 41 (6.2) | T8523A | 40 (6.1) | 16 (39) | 16 (40) | 0.35535847 | 1.30126E-10 |
| T23617G | 320 (48.7) | C3634A | 79 (12) | 77 (24.1) | 77 (97.5) | 0.360667975 | 1.68681E-23 |
| G4953A | 47 (7.2) | T7904A | 38 (5.8) | 17 (36.2) | 17 (44.7) | 0.361318956 | 4.03494E-11 |
| T14537A | 39 (5.9) | T8166A | 36 (5.5) | 15 (38.5) | 15 (41.7) | 0.364069678 | 1.31336E-10 |
| C5672A | 184 (28) | C1902A | 35 (5.3) | 34 (18.5) | 34 (97.1) | 0.365236184 | 1.28083E-18 |
| T23617G | 320 (48.7) | C14714A | 81 (12.3) | 79 (24.7) | 79 (97.5) | 0.366307324 | 3.15653E-24 |
| A27259C | 488 (74.3) | C5672A | 184 (28) | 184 (37.7) | 184 (100) | 0.367038636 | 8.43823E-29 |
| T10234A | 41 (6.2) | T7904A | 38 (5.8) | 16 (39) | 16 (42.1) | 0.36736492 | 5.01952E-11 |
| T22917G | 164 (25) | C26577G | 461 (70.2) | 163 (99.4) | 163 (35.4) | 0.368389635 | 5.39175E-28 |
| A23040G | 487 (74.1) | C5672A | 184 (28) | 184 (37.8) | 184 (100) | 0.368500703 | 7.12543E-29 |
| C15080A | 130 (19.8) | T23620G | 334 (50.8) | 115 (88.5) | 115 (34.4) | 0.373789266 | 3.83016E-23 |
| T23620G | 334 (50.8) | C23613G | 147 (22.4) | 126 (37.7) | 126 (85.7) | 0.374546212 | 1.234E-22 |
| T8523A | 40 (6.1) | G20111A | 42 (6.4) | 17 (42.5) | 17 (40.5) | 0.375825 | 1.27783E-11 |
| G12160A | 170 (25.9) | C26577G | 461 (70.2) | 169 (99.4) | 169 (36.7) | 0.377649254 | 4.40018E-29 |
| G26529A | 170 (25.9) | C26577G | 461 (70.2) | 169 (99.4) | 169 (36.7) | 0.377649254 | 4.40018E-29 |
| T23018G | 170 (25.9) | C26577G | 461 (70.2) | 169 (99.4) | 169 (36.7) | 0.377649254 | 4.40018E-29 |
| T14537A | 39 (5.9) | T7904A | 38 (5.8) | 16 (41) | 16 (42.1) | 0.379248687 | 1.98729E-11 |
| C10647A | 35 (5.3) | C27154A | 164 (25) | 33 (94.3) | 33 (20.1) | 0.379962699 | 1.13637E-18 |
| C10647A | 35 (5.3) | C5672A | 184 (28) | 35 (100) | 35 (19) | 0.380329917 | 1.21885E-20 |
| G5529A | 39 (5.9) | G20263A | 41 (6.2) | 17 (43.6) | 17 (41.5) | 0.387885113 | 4.88013E-12 |
| T14537A | 39 (5.9) | G20263A | 41 (6.2) | 17 (43.6) | 17 (41.5) | 0.387885113 | 4.88013E-12 |
| T23617G | 320 (48.7) | C24876A | 89 (13.5) | 87 (27.2) | 87 (97.8) | 0.38842296 | 3.60381E-27 |
| A27259C | 488 (74.3) | T23620G | 334 (50.8) | 304 (62.3) | 304 (91) | 0.38946112 | 5.73699E-24 |
| C10647A | 35 (5.3) | C19042A | 167 (25.4) | 34 (97.1) | 34 (20.4) | 0.39076366 | 3.62375E-20 |
| C10647A | 35 (5.3) | C3634A | 79 (12) | 23 (65.7) | 23 (29.1) | 0.391579181 | 2.41737E-14 |
| C3634A | 79 (12) | C1902A | 35 (5.3) | 23 (29.1) | 23 (65.7) | 0.391579181 | 2.41737E-14 |
| A23040G | 487 (74.1) | T23620G | 334 (50.8) | 304 (62.4) | 304 (91) | 0.392246566 | 2.64636E-24 |
| G5529A | 39 (5.9) | T8166A | 36 (5.5) | 16 (41) | 16 (44.4) | 0.392373285 | 7.18215E-12 |
| G5529A | 39 (5.9) | T8523A | 40 (6.1) | 17 (43.6) | 17 (42.5) | 0.393984444 | 2.96582E-12 |
| T14537A | 39 (5.9) | T20529A | 44 (6.7) | 18 (46.2) | 18 (40.9) | 0.396522686 | 1.124E-12 |
| G20111A | 42 (6.4) | T16548A | 40 (6.1) | 18 (42.9) | 18 (45) | 0.401846396 | 7.35636E-13 |
| C11871A | 48 (7.3) | C1902A | 35 (5.3) | 18 (37.5) | 18 (51.4) | 0.402194733 | 6.26951E-13 |
| T7904A | 38 (5.8) | T19427A | 44 (6.7) | 18 (47.4) | 18 (40.9) | 0.403128461 | 6.43575E-13 |
| T11709A | 45 (6.8) | T10234A | 41 (6.2) | 19 (42.2) | 19 (46.3) | 0.403362698 | 3.10382E-13 |
| C1902A | 35 (5.3) | C19042A | 167 (25.4) | 35 (100) | 35 (21) | 0.406329762 | 2.89344E-22 |
| C14292A | 72 (11) | C11871A | 48 (7.3) | 27 (37.5) | 27 (56.3) | 0.407048342 | 1.08178E-15 |
| T10234A | 41 (6.2) | T16548A | 40 (6.1) | 18 (43.9) | 18 (45) | 0.407989334 | 4.37887E-13 |
| G20263A | 41 (6.2) | T19427A | 44 (6.7) | 19 (46.3) | 19 (43.2) | 0.40915864 | 1.86889E-13 |
| C27154A | 164 (25) | C1902A | 35 (5.3) | 35 (21.3) | 35 (100) | 0.411282628 | 1.45519E-22 |
| T10234A | 41 (6.2) | G5529A | 39 (5.9) | 18 (43.9) | 18 (46.2) | 0.414514217 | 2.54903E-13 |
| T14537A | 39 (5.9) | T10234A | 41 (6.2) | 18 (46.2) | 18 (43.9) | 0.414514217 | 2.54903E-13 |
| C15485A | 162 (24.7) | C10647A | 35 (5.3) | 35 (21.6) | 35 (100) | 0.41465215 | 9.17759E-23 |
| C15485A | 162 (24.7) | C1902A | 35 (5.3) | 35 (21.6) | 35 (100) | 0.41465215 | 9.17759E-23 |
| T19427A | 44 (6.7) | T20529A | 44 (6.7) | 20 (45.5) | 20 (45.5) | 0.415393742 | 5.29208E-14 |
| C11871A | 48 (7.3) | C5140A | 44 (6.7) | 21 (43.8) | 21 (47.7) | 0.416143271 | 2.36708E-14 |
| T8166A | 36 (5.5) | T19427A | 44 (6.7) | 18 (50) | 18 (40.9) | 0.417091314 | 1.93744E-13 |
| G22578A | 192 (29.2) | C26577G | 461 (70.2) | 192 (100) | 192 (41.6) | 0.418988122 | 3.04787E-36 |
| T23620G | 334 (50.8) | C27154A | 164 (25) | 143 (42.8) | 143 (87.2) | 0.419458623 | 2.02661E-28 |
| T7904A | 38 (5.8) | T6074A | 50 (7.6) | 20 (52.6) | 20 (40) | 0.420678039 | 2.87384E-14 |
| T7904A | 38 (5.8) | G20263A | 41 (6.2) | 18 (47.4) | 18 (43.9) | 0.421275743 | 1.4556E-13 |
| T10234A | 41 (6.2) | G20111A | 42 (6.4) | 19 (46.3) | 19 (45.2) | 0.421316186 | 6.56124E-14 |
| G4953A | 47 (7.2) | T19427A | 44 (6.7) | 21 (44.7) | 21 (47.7) | 0.42178445 | 1.38634E-14 |
| G4953A | 47 (7.2) | T8166A | 36 (5.5) | 19 (40.4) | 19 (52.8) | 0.426236656 | 3.84271E-14 |
| T23620G | 334 (50.8) | C19042A | 167 (25.4) | 146 (43.7) | 146 (87.4) | 0.427257676 | 1.75926E-29 |
| T7904A | 38 (5.8) | T16548A | 40 (6.1) | 18 (47.4) | 18 (45) | 0.427740564 | 8.50436E-14 |
| G20111A | 42 (6.4) | T19427A | 44 (6.7) | 20 (47.6) | 20 (45.5) | 0.427811142 | 1.68535E-14 |
| C15485A | 162 (24.7) | T23620G | 334 (50.8) | 143 (88.3) | 143 (42.8) | 0.428368404 | 9.11681E-30 |
| C14292A | 72 (11) | C26885A | 124 (18.9) | 48 (66.7) | 48 (38.7) | 0.428492787 | 2.99934E-21 |
| C5672A | 184 (28) | C5140A | 44 (6.7) | 44 (23.9) | 44 (100) | 0.429553992 | 2.99899E-26 |
| A9424G | 315 (47.9) | C26577G | 461 (70.2) | 286 (90.8) | 286 (62) | 0.432662052 | 8.73732E-30 |
| T11709A | 45 (6.8) | T19427A | 44 (6.7) | 21 (46.7) | 21 (47.7) | 0.433579452 | 4.55352E-15 |
| T8523A | 40 (6.1) | G20263A | 41 (6.2) | 19 (47.5) | 19 (46.3) | 0.434304766 | 2.12101E-14 |
| G5529A | 39 (5.9) | G20111A | 42 (6.4) | 19 (48.7) | 19 (45.2) | 0.434651129 | 2.04077E-14 |
| T8166A | 36 (5.5) | T6074A | 50 (7.6) | 20 (55.6) | 20 (40) | 0.435348103 | 6.99E-15 |
| T10234A | 41 (6.2) | T8166A | 36 (5.5) | 18 (43.9) | 18 (50) | 0.435573064 | 4.36567E-14 |
| C11871A | 48 (7.3) | C5672A | 184 (28) | 47 (97.9) | 47 (25.5) | 0.437099948 | 1.31855E-26 |
| T20529A | 44 (6.7) | T16548A | 40 (6.1) | 20 (45.5) | 20 (50) | 0.441075676 | 4.95471E-15 |
| T8523A | 40 (6.1) | T20529A | 44 (6.7) | 20 (50) | 20 (45.5) | 0.441075676 | 4.95471E-15 |
| T10234A | 41 (6.2) | G4953A | 47 (7.2) | 21 (51.2) | 21 (44.7) | 0.441117805 | 2.14302E-15 |
| T7904A | 38 (5.8) | G20111A | 42 (6.4) | 19 (50) | 19 (45.2) | 0.441681183 | 1.09439E-14 |
| T11709A | 45 (6.8) | G5529A | 39 (5.9) | 20 (44.4) | 20 (51.3) | 0.441900534 | 4.50134E-15 |
| T14537A | 39 (5.9) | T11709A | 45 (6.8) | 20 (51.3) | 20 (44.4) | 0.441900534 | 4.50134E-15 |
| T8166A | 36 (5.5) | T16548A | 40 (6.1) | 18 (50) | 18 (45) | 0.442159225 | 2.53225E-14 |
| T8166A | 36 (5.5) | T20529A | 44 (6.7) | 19 (52.8) | 19 (43.2) | 0.443846732 | 8.56922E-15 |
| G23642T | 51 (7.8) | G28936T | 95 (14.5) | 35 (68.6) | 35 (36.8) | 0.446812453 | 3.7484E-20 |
| C11871A | 48 (7.3) | C26885A | 124 (18.9) | 39 (81.3) | 39 (31.5) | 0.44753059 | 3.52771E-22 |
| T14537A | 39 (5.9) | T16548A | 40 (6.1) | 19 (48.7) | 19 (47.5) | 0.447860556 | 6.47574E-15 |
| T14537A | 39 (5.9) | T8523A | 40 (6.1) | 19 (48.7) | 19 (47.5) | 0.447860556 | 6.47574E-15 |
| T23617G | 320 (48.7) | C26885A | 124 (18.9) | 118 (36.9) | 118 (95.2) | 0.448286436 | 2.08059E-34 |
| G22578A | 192 (29.2) | G23642T | 51 (7.8) | 51 (26.6) | 51 (100) | 0.451465573 | 1.16379E-29 |
| T14537A | 39 (5.9) | G5529A | 39 (5.9) | 19 (48.7) | 19 (48.7) | 0.454817028 | 3.53498E-15 |
| T6074A | 50 (7.6) | T16548A | 40 (6.1) | 22 (44) | 22 (55) | 0.455047713 | 2.36666E-16 |
| T23617G | 320 (48.7) | C15080A | 130 (19.8) | 123 (38.4) | 123 (94.6) | 0.456184994 | 2.51544E-35 |
| C14714A | 81 (12.3) | C26885A | 124 (18.9) | 54 (66.7) | 54 (43.5) | 0.458022137 | 2.57189E-24 |
| C5140A | 44 (6.7) | C19042A | 167 (25.4) | 44 (100) | 44 (26.3) | 0.458918859 | 2.3168E-28 |
| T11709A | 45 (6.8) | T16548A | 40 (6.1) | 21 (46.7) | 21 (52.5) | 0.4601698 | 3.58627E-16 |
| C15173A | 42 (6.4) | G15168A | 89 (13.5) | 31 (73.8) | 31 (34.8) | 0.460187438 | 3.34809E-20 |
| C15080A | 130 (19.8) | C10647A | 35 (5.3) | 34 (26.2) | 34 (97.1) | 0.460596806 | 2.76115E-24 |
| G5529A | 39 (5.9) | T7904A | 38 (5.8) | 19 (48.7) | 19 (50) | 0.462028218 | 1.87427E-15 |
| T23620G | 334 (50.8) | C5672A | 184 (28) | 162 (48.5) | 162 (88) | 0.464179722 | 9.85516E-35 |
| C5140A | 44 (6.7) | C27154A | 164 (25) | 44 (100) | 44 (26.8) | 0.464512747 | 9.25346E-29 |
| T6074A | 50 (7.6) | G20111A | 42 (6.4) | 23 (46) | 23 (54.8) | 0.464696205 | 4.08189E-17 |
| C15485A | 162 (24.7) | C5140A | 44 (6.7) | 44 (27.2) | 44 (100) | 0.46831837 | 4.96998E-29 |
| G4953A | 47 (7.2) | T20529A | 44 (6.7) | 23 (48.9) | 23 (52.3) | 0.469036966 | 2.79983E-17 |
| T23617G | 320 (48.7) | C23613G | 147 (22.4) | 136 (42.5) | 136 (92.5) | 0.470576092 | 1.64867E-36 |
| G20263A | 41 (6.2) | G20111A | 42 (6.4) | 21 (51.2) | 21 (50) | 0.472762099 | 1.133E-16 |
| C15485A | 162 (24.7) | C11871A | 48 (7.3) | 47 (29) | 47 (97.9) | 0.477176455 | 1.4015E-29 |
| T20529A | 44 (6.7) | G20111A | 42 (6.4) | 22 (50) | 22 (52.4) | 0.477593628 | 2.85585E-17 |
| C15080A | 130 (19.8) | C1902A | 35 (5.3) | 35 (26.9) | 35 (100) | 0.477608955 | 1.49347E-26 |
| G5529A | 39 (5.9) | G4953A | 47 (7.2) | 22 (56.4) | 22 (46.8) | 0.48012396 | 2.05386E-17 |
| T14537A | 39 (5.9) | G4953A | 47 (7.2) | 22 (56.4) | 22 (46.8) | 0.48012396 | 2.05386E-17 |
| C11871A | 48 (7.3) | C19042A | 167 (25.4) | 48 (100) | 48 (28.7) | 0.480896678 | 3.8214E-31 |
| C5140A | 44 (6.7) | C3634A | 79 (12) | 31 (70.5) | 31 (39.2) | 0.481306051 | 4.35162E-21 |
| G5529A | 39 (5.9) | T6074A | 50 (7.6) | 23 (59) | 23 (46) | 0.486612208 | 3.97141E-18 |
| C11871A | 48 (7.3) | C27154A | 164 (25) | 48 (100) | 48 (29.3) | 0.48675846 | 1.41154E-31 |
| C10647A | 35 (5.3) | C1902A | 35 (5.3) | 18 (51.4) | 18 (51.4) | 0.486954525 | 6.29612E-16 |
| C11871A | 48 (7.3) | C3634A | 79 (12) | 34 (70.8) | 34 (43) | 0.489642757 | 4.79135E-22 |
| T11709A | 45 (6.8) | T6074A | 50 (7.6) | 25 (55.6) | 25 (50) | 0.49029984 | 5.37076E-19 |
| T8166A | 36 (5.5) | G20263A | 41 (6.2) | 20 (55.6) | 20 (48.8) | 0.490871905 | 5.3549E-17 |
| T19427A | 44 (6.7) | T16548A | 40 (6.1) | 22 (50) | 22 (55) | 0.492004801 | 6.80334E-18 |
| T15474G | 42 (6.4) | G28936T | 95 (14.5) | 34 (81) | 34 (35.8) | 0.494079196 | 1.51658E-23 |
| G4953A | 47 (7.2) | T16548A | 40 (6.1) | 23 (48.9) | 23 (57.5) | 0.497400532 | 1.4616E-18 |
| G5529A | 39 (5.9) | T19427A | 44 (6.7) | 22 (56.4) | 22 (50) | 0.499595051 | 3.17687E-18 |
| C24876A | 89 (13.5) | C26885A | 124 (18.9) | 61 (68.5) | 61 (49.2) | 0.502421191 | 3.41038E-29 |
| T8523A | 40 (6.1) | T6074A | 50 (7.6) | 24 (60) | 24 (48) | 0.50305901 | 2.98852E-19 |
| C15080A | 130 (19.8) | C11871A | 48 (7.3) | 44 (33.8) | 44 (91.7) | 0.50653208 | 5.43797E-29 |
| G4953A | 47 (7.2) | G20111A | 42 (6.4) | 24 (51.1) | 24 (57.1) | 0.506890524 | 2.2433E-19 |
| T11709A | 45 (6.8) | G4953A | 47 (7.2) | 25 (55.6) | 25 (53.2) | 0.509264988 | 7.14758E-20 |
| T10234A | 41 (6.2) | T19427A | 44 (6.7) | 23 (56.1) | 23 (52.3) | 0.509848677 | 4.45111E-19 |
| T10234A | 41 (6.2) | T20529A | 44 (6.7) | 23 (56.1) | 23 (52.3) | 0.509848677 | 4.45111E-19 |
| C14292A | 72 (11) | C5140A | 44 (6.7) | 31 (43.1) | 31 (70.5) | 0.510272824 | 1.37226E-22 |
| T8523A | 40 (6.1) | T11709A | 45 (6.8) | 23 (57.5) | 23 (51.1) | 0.510570993 | 4.02709E-19 |
| G4953A | 47 (7.2) | G20263A | 41 (6.2) | 24 (51.1) | 24 (58.5) | 0.514364915 | 1.01761E-19 |
| C3634A | 79 (12) | C26885A | 124 (18.9) | 58 (73.4) | 58 (46.8) | 0.515333004 | 3.83926E-30 |
| T6074A | 50 (7.6) | T20529A | 44 (6.7) | 26 (52) | 26 (59.1) | 0.520146136 | 8.03124E-21 |
| T11709A | 45 (6.8) | G20111A | 42 (6.4) | 24 (53.3) | 24 (57.1) | 0.520334608 | 5.69209E-20 |
| T11709A | 45 (6.8) | G20263A | 41 (6.2) | 24 (53.3) | 24 (58.5) | 0.527920554 | 2.53164E-20 |
| T23617G | 320 (48.7) | C27154A | 164 (25) | 155 (48.4) | 155 (94.5) | 0.528561873 | 1.59301E-46 |
| T11709A | 45 (6.8) | T20529A | 44 (6.7) | 25 (55.6) | 25 (56.8) | 0.530003824 | 7.59132E-21 |
| T23617G | 320 (48.7) | C15485A | 162 (24.7) | 154 (48.1) | 154 (95.1) | 0.530556323 | 3.16923E-47 |
| C5140A | 44 (6.7) | C1902A | 35 (5.3) | 22 (50) | 22 (62.9) | 0.532935919 | 1.09839E-19 |
| T8166A | 36 (5.5) | G20111A | 42 (6.4) | 22 (61.1) | 22 (52.4) | 0.53856998 | 7.09368E-20 |
| C15080A | 130 (19.8) | C5140A | 44 (6.7) | 44 (33.8) | 44 (100) | 0.539423338 | 5.67997E-34 |
| C14292A | 72 (11) | C5672A | 184 (28) | 70 (97.2) | 70 (38) | 0.540781145 | 2.18914E-40 |
| T8523A | 40 (6.1) | T19427A | 44 (6.7) | 24 (60) | 24 (54.5) | 0.542933926 | 5.25836E-21 |
| T23617G | 320 (48.7) | C19042A | 167 (25.4) | 59 (18.4) | 59 (35.3) | 0.543149989 | 1.93372E-49 |
| T11709A | 45 (6.8) | T8166A | 36 (5.5) | 23 (51.1) | 23 (63.9) | 0.54370725 | 1.24801E-20 |
| G4953A | 47 (7.2) | T8523A | 40 (6.1) | 25 (53.2) | 25 (62.5) | 0.546798484 | 1.10942E-21 |
| T8523A | 40 (6.1) | T16548A | 40 (6.1) | 23 (57.5) | 23 (57.5) | 0.547447326 | 1.0332E-20 |
| G5529A | 39 (5.9) | T20529A | 44 (6.7) | 24 (61.5) | 24 (54.5) | 0.551131234 | 2.17781E-21 |
| T14537A | 39 (5.9) | T19427A | 44 (6.7) | 24 (61.5) | 24 (54.5) | 0.551131234 | 2.17781E-21 |
| T10234A | 41 (6.2) | G20263A | 41 (6.2) | 24 (58.5) | 24 (58.5) | 0.557768451 | 1.15551E-21 |
| T14537A | 39 (5.9) | T6074A | 50 (7.6) | 26 (66.7) | 26 (52) | 0.559487572 | 8.22873E-23 |
| T7904A | 38 (5.8) | T20529A | 44 (6.7) | 24 (63.2) | 24 (54.5) | 0.559631553 | 8.70114E-22 |
| C24876A | 89 (13.5) | C10647A | 35 (5.3) | 33 (37.1) | 33 (94.3) | 0.559654449 | 1.10456E-28 |
| T20529A | 44 (6.7) | G20263A | 41 (6.2) | 25 (56.8) | 25 (61) | 0.560193696 | 2.8557E-22 |
| G4953A | 47 (7.2) | T6074A | 50 (7.6) | 29 (61.7) | 29 (58) | 0.566240967 | 2.05167E-24 |
| C14714A | 81 (12.3) | C11871A | 48 (7.3) | 38 (46.9) | 38 (79.2) | 0.57075061 | 3.31061E-29 |
| C15080A | 130 (19.8) | C14292A | 72 (11) | 71 (54.6) | 71 (98.6) | 0.57181775 | 3.92138E-37 |
| C5672A | 184 (28) | C3634A | 79 (12) | 77 (41.8) | 77 (97.5) | 0.571905748 | 2.48066E-45 |
| A27259C | 488 (74.3) | T23617G | 320 (48.7) | 320 (65.6) | 320 (100) | 0.573447257 | 6.43914E-61 |
| C14292A | 72 (11) | C3634A | 79 (12) | 47 (65.3) | 47 (59.5) | 0.574412341 | 1.20517E-31 |
| C14292A | 72 (11) | C27154A | 164 (25) | 69 (95.8) | 69 (42.1) | 0.574485698 | 9.98686E-43 |
| A23040G | 487 (74.1) | T23617G | 320 (48.7) | 320 (65.7) | 320 (100) | 0.575731536 | 2.17649E-61 |
| C15485A | 162 (24.7) | C14292A | 72 (11) | 69 (42.6) | 69 (95.8) | 0.579329881 | 3.37996E-43 |
| C24876A | 89 (13.5) | C1902A | 35 (5.3) | 34 (38.2) | 34 (97.1) | 0.57945909 | 6.72375E-31 |
| C14714A | 81 (12.3) | C5672A | 184 (28) | 79 (97.5) | 79 (42.9) | 0.580626366 | 9.11916E-47 |
| T6074A | 50 (7.6) | T19427A | 44 (6.7) | 29 (58) | 29 (65.9) | 0.589035267 | 1.18024E-25 |
| C24876A | 89 (13.5) | C11871A | 48 (7.3) | 41 (46.1) | 41 (85.4) | 0.589599745 | 2.74449E-32 |
| C14292A | 72 (11) | C19042A | 167 (25.4) | 71 (98.6) | 71 (42.5) | 0.589744961 | 2.42334E-46 |
| C14714A | 81 (12.3) | C10647A | 35 (5.3) | 33 (40.7) | 33 (94.3) | 0.59133859 | 2.39317E-30 |
| T23617G | 320 (48.7) | C5672A | 184 (28) | 177 (55.3) | 177 (96.2) | 0.592584872 | 1.80697E-59 |
| T14537A | 39 (5.9) | G20111A | 42 (6.4) | 25 (64.1) | 25 (59.5) | 0.592640502 | 8.85971E-24 |
| C14714A | 81 (12.3) | C14292A | 72 (11) | 49 (60.5) | 49 (68.1) | 0.594653458 | 6.196E-34 |
| C14714A | 81 (12.3) | C1902A | 35 (5.3) | 34 (42) | 34 (97.1) | 0.611953545 | 1.25521E-32 |
| T10234A | 41 (6.2) | T6074A | 50 (7.6) | 29 (70.7) | 29 (58) | 0.614135104 | 4.61684E-27 |
| T6074A | 50 (7.6) | G20263A | 41 (6.2) | 29 (58) | 29 (70.7) | 0.614135104 | 4.61684E-27 |
| C15485A | 162 (24.7) | C3634A | 79 (12) | 77 (47.5) | 77 (97.5) | 0.624526416 | 7.08529E-51 |
| C24876A | 89 (13.5) | C5672A | 184 (28) | 88 (98.9) | 88 (47.8) | 0.624756947 | 5.56263E-55 |
| C3634A | 79 (12) | C27154A | 164 (25) | 78 (98.7) | 78 (47.6) | 0.630176956 | 1.18836E-52 |
| C14714A | 81 (12.3) | C19042A | 167 (25.4) | 80 (98.8) | 80 (47.9) | 0.631716041 | 1.91388E-53 |
| C3634A | 79 (12) | C19042A | 167 (25.4) | 79 (100) | 79 (47.3) | 0.633270753 | 1.82806E-54 |
| C14714A | 81 (12.3) | C27154A | 164 (25) | 80 (98.8) | 80 (48.8) | 0.639481694 | 2.68039E-54 |
| C10647A | 35 (5.3) | C5140A | 44 (6.7) | 26 (74.3) | 26 (59.1) | 0.64138842 | 8.15685E-27 |
| C15485A | 162 (24.7) | C14714A | 81 (12.3) | 81 (50) | 81 (100) | 0.65550553 | 1.44514E-57 |
| C24876A | 89 (13.5) | C14292A | 72 (11) | 56 (62.9) | 56 (77.8) | 0.658463331 | 3.29668E-42 |
| C24876A | 89 (13.5) | C5140A | 44 (6.7) | 43 (48.3) | 43 (97.7) | 0.659030636 | 1.95698E-40 |
| C24876A | 89 (13.5) | C3634A | 79 (12) | 59 (66.3) | 59 (74.7) | 0.660466523 | 2.84215E-43 |
| C24876A | 89 (13.5) | C27154A | 164 (25) | 87 (97.8) | 87 (53) | 0.665759966 | 1.28073E-58 |
| C15080A | 130 (19.8) | C3634A | 79 (12) | 73 (56.2) | 73 (92.4) | 0.673881 | 2.08525E-52 |
| C14714A | 81 (12.3) | C5140A | 44 (6.7) | 42 (51.9) | 42 (95.5) | 0.677397273 | 2.32228E-40 |
| C24876A | 89 (13.5) | C19042A | 167 (25.4) | 89 (100) | 89 (53.3) | 0.678048313 | 8.11561E-63 |
| C15485A | 162 (24.7) | C24876A | 89 (13.5) | 88 (54.3) | 88 (98.9) | 0.681617038 | 9.97478E-62 |
| C14714A | 81 (12.3) | C3634A | 79 (12) | 58 (71.6) | 58 (73.4) | 0.686948116 | 2.15578E-45 |
| C15080A | 130 (19.8) | C26885A | 124 (18.9) | 96 (73.8) | 96 (77.4) | 0.697754531 | 5.97382E-59 |
| C15080A | 130 (19.8) | C14714A | 81 (12.3) | 77 (59.2) | 77 (95.1) | 0.708548151 | 1.31938E-58 |
| C24876A | 89 (13.5) | C15080A | 130 (19.8) | 82 (92.1) | 82 (63.1) | 0.718845037 | 2.36735E-60 |
| G23642T | 51 (7.8) | T15474G | 42 (6.4) | 35 (68.6) | 35 (83.3) | 0.738048565 | 5.67272E-38 |
| C5672A | 184 (28) | C26885A | 124 (18.9) | 120 (65.2) | 120 (96.8) | 0.738686538 | 6.6063E-77 |
| C15080A | 130 (19.8) | C5672A | 184 (28) | 126 (96.9) | 126 (68.5) | 0.762287062 | 1.9565E-82 |
| T23620G | 334 (50.8) | T23617G | 320 (48.7) | 288 (86.2) | 288 (90) | 0.763352848 | 1.04714E-94 |
| C27154A | 164 (25) | C26885A | 124 (18.9) | 118 (72) | 118 (95.2) | 0.782348521 | 3.04208E-82 |
| C15485A | 162 (24.7) | C26885A | 124 (18.9) | 120 (74.1) | 120 (96.8) | 0.807027068 | 4.16887E-88 |
| C15080A | 130 (19.8) | C27154A | 164 (25) | 124 (95.4) | 124 (75.6) | 0.80816274 | 1.28844E-88 |
| C19042A | 167 (25.4) | C26885A | 124 (18.9) | 122 (73.1) | 122 (98.4) | 0.808335988 | 1.96979E-90 |
| C15485A | 162 (24.7) | C15080A | 130 (19.8) | 125 (77.2) | 125 (96.2) | 0.823863516 | 2.87901E-92 |
| C15080A | 130 (19.8) | C19042A | 167 (25.4) | 129 (99.2) | 129 (77.2) | 0.841983561 | 7.0847E-100 |
| C5672A | 184 (28) | C19042A | 167 (25.4) | 158 (85.9) | 158 (94.6) | 0.86594617 | 3.7788E-110 |
| C24876A | 89 (13.5) | C14714A | 81 (12.3) | 75 (84.3) | 75 (92.6) | 0.866180286 | 1.62373E-75 |
| C5672A | 184 (28) | C27154A | 164 (25) | 157 (85.3) | 157 (95.7) | 0.869916033 | 1.9149E-111 |
| C15485A | 162 (24.7) | C5672A | 184 (28) | 156 (96.3) | 156 (84.8) | 0.870040023 | 1.4742E-111 |
| C15485A | 162 (24.7) | C27154A | 164 (25) | 153 (94.4) | 153 (93.3) | 0.918438977 | 1.8125E-122 |
| C19042A | 167 (25.4) | C27154A | 164 (25) | 156 (93.4) | 156 (95.1) | 0.923339487 | 4.3201E-125 |
| C15485A | 162 (24.7) | C19042A | 167 (25.4) | 158 (97.5) | 158 (94.6) | 0.947487128 | 5.418E-134 |

^a^ Covariation frequency based on the prevalence of SNP 1.

^b^ Covariation frequency based on the prevalence of SNP 2.

^c^ All P-values for covariation were significant at a false discovery rate of 0.05.

SNP: single nucleotide polymorphism
